# Discriminative Prior - Prior Image Constrained Compressed Sensing Reconstruction for Low-Dose CT Imaging

**DOI:** 10.1038/s41598-017-13520-y

**Published:** 2017-10-24

**Authors:** Yang Chen, Jin Liu, Lizhe Xie, Yining Hu, Huazhong Shu, Limin Luo, Libo Zhang, Zhiguo Gui, Gouenou Coatrieux

**Affiliations:** 1 Laboratory of Image Science and Technology, School of Computer Science and Engineering, Southeast University, Nanjing, 210096 China; 2 Key Laboratory of Computer Network and Information Integration (Southeast University), Ministry of Education, Nanjing, 210096 China; 3 International Joint Research Laboratory of Information Display and Visualization, Southeast University, Ministry of Education, Nanjing, 210096 China; 4 Jiangsu Key Laboratory of Oral Diseases, Nanjing Medical University, No. 140, Hanzhong road, Nanjing, 210000 China; 5 The General Hospital of Shenyang Military, Shenyang, Liaoning 110016 China; 6 The Institut Mines-Telecom, Telecom Bretagne; INSERM U1101 LaTIM, Brest, 29238 France; 7 The National Key Laboratory for Electronic Measurement Technology, North University of China, Taiyuan, 030051 China

## Abstract

X-ray computed tomography (CT) has been widely used to provide patient-specific anatomical information in the forms of tissue attenuation. However, the cumulative radiation induced in CT scan has raised extensive concerns in recently years. How to maintain reconstruction image quality is a major challenge for low-dose CT (LDCT) imaging. Generally, LDCT imaging can be greatly improved by incorporating prior knowledge in some specific forms. A joint estimation framework termed discriminative prior-prior image constrained compressed sensing (DP-PICCS) reconstruction is proposed in this paper. This DP-PICCS algorithm utilizes discriminative prior knowledge via two feature dictionary constraints which built on atoms from the samples of tissue attenuation feature patches and noise-artifacts residual feature patches, respectively. Also, the prior image construction relies on a discriminative feature representation (DFR) processing by two feature dictionary. Its comparison to other competing methods through experiments on low-dose projections acquired from torso phantom simulation study and clinical abdomen study demonstrated that the DP-PICCS method achieved promising improvement in terms of the effectively-suppressed noise and the well-retained structures.

## Introduction

X-ray computed tomography (CT) is widely used in diagnostic imaging, image-guided surgeries and radiotherapy tasks for clinic applications^1–3^. Recent years, more and more attention has been paid to the risk of ionizing radiation received during CT scans in recent years. CT dose levels should be kept ALARA (as low as reasonably achievable) if enough diagnostic information is provided. Reducing the exposure level (low-exposure protocol) can be simply implemented to lower doses in clinic application^4–6^. Conventional analytical reconstruction algorithms (*e.g*. Filter Back Projection (FBP) method, Feldkamp-Davis-Kress (FDK) method^7^) often lead to unsatisfactory diagnostic CT image quality with increased mottle noise and streak artifacts due to the deteriorated projections.

Derived from statistical models of projection data, iterative methods have demonstrated better performance in noise-artifacts suppression in reconstruction, and lead to improved image quality. With the rapid development of compressed sensing (CS)^8,9^, sparsity-promotion (*e.g*., total variation (TV)^10–13^, tight frame (TF)^14^, wavelet transform^15^, and dictionary learning^16–23^) based constraint had been considered to alleviate the ill-posedness in low-dose CT (LDCT) reconstruction. For example, the TV minimization based constraint (or regularization) reconstruction, which was developed based on the sparse distribution of discrete gradient. Such constraint has demonstrated its effectiveness in preserving edges and suppressing noise^11–23^. Recently, dictionary based sparse representation was introduced to improve medical imaging^16–23^. In^16^, Xu. *et al*. proposed a dictionary learning (DL) based method for LDCT reconstruction and demonstrated performance superior to the TV based method. Containing rich feature information, the composing atoms in dictionary work well in representing structured features, which lead to significantly enhanced reconstruction quality. In^19^, Liu. *et al*. proposed an iterative reconstruction by feature constrained to improve LDCT imaging, which relies on a pre-defined 3D feature dictionary constructed from standard-dose CT (SDCT) sample. The pre-defined 3D feature dictionary contains patient-specific anatomical structures, which have significant morphological discrepancy with the undesirable noise-artifacts features. This 3D feature constraint approach has good potential for suppressed noise-artifacts and the better retained anatomical structures.

Prior knowledge can also be utilized by directly including some available high quality prior image into the cost function, which leads to the algorithms termed prior image constrained compressed sensing (PICCS)^24^. Some PICCS type algorithms have gotten successful applications in interventional imaging, treatment monitoring, and 4D cardiac reconstruction^24–27^. Nevertheless, the prior image term takes effect in PICCS algorithms via the minimization of subtracted residual function, which requires an exact position correspondence between the prior image and the current image. This requirement, however, is often hard to meet due to the unavailability of precisely matched high quality prior images, which often greatly limits the practical feasibility.

This paper presents a reconstruction approach termed discriminative prior - prior image constrained compressed sensing (DP-PICCS) for LDCT, which improves current PICCS reconstruction by utilizing discriminative prior knowledge. The discriminative prior knowledge works through feature dictionaries which containing rich tissue attenuation feature information and noise-artifacts residual feature information. The proposed DP-PICCS algorithm can be easily implemented via an alternative optimization scheme with a good parameter robustness. Experiments with simulated torso phantom and clinical abdomen data were conducted though the comparison with three state-of-art reconstruction algorithms. In summary, this paper is structured as follows: in section II, we present a detailed description of the proposed method and the implementation procedure for LDCT reconstruction. Experimental results and quantify the performance from simulated torso phantom and clinical abdomen projection are given and parameters setting of our proposed method are discussed in section III. Finally, conclusions the paper and plans for future work are sketched in section IV.

## M**ethod**

### Discriminative prior - prior image constrained compressed sensing model (DP-PICCS)

The standard CT reconstruction problem can be considered as an inverse problem. Directly solving the it may not be feasible due to the measurement incompletion or noise contamination in projection data. Approaches based on sparse representation can be used to overcome the ill-poseness by incorporating representation related knowledge in the form of a regularization term^11,28^. The formula for CT image reconstruction with a constraint term can be expressed as:

1 $$\begin{array}{c}{u}^{\ast }=\text{arg}\,\mathop{\min }\limits_{u}\lambda {R}_{g}(u)\,\\ s.t.{(Gu-p)}^{T}W(Gu-p)=0\end{array}$$

where $G=\{{g}_{mn}\}\in {R}^{M\times N}$ is the system matrix representing the contribution of the *n-*th voxel to the *m-*th X-ray path; $u=\{{u}_{n}\}\in {R}^{N}$ denotes the discrete vector of target image reconstructed from the projection data $p=\{{p}_{m}\}\in {R}^{M}$. *N* is the total voxel number of the volume to reconstruct; *W* is projection noise statistics weight matrix, *R*_*g*_ denotes some specific global constraint term. Specifically, further reconstruction improvement can be brought via the PICCS approach by directly incorporating a high-quality prior image ${u}_{{\rm{prior}}}$ into cost function^24^. This is accomplished by incorporating an image similar to that which we want to reconstruct into the reconstruction procedure. The PICCS algorithm can be formulated as a constrained optimization procedure as follows:

2 $$\begin{array}{c}{u}^{\ast }=\text{arg}\mathop{\min }\limits_{u}\,[\lambda {R}_{p}(u-{u}_{{\rm{prior}}})+(1-\lambda ){R}_{g}(u)]\\ {s}{\rm{.}}{t}{\rm{.}}\,\,{(Gu-p)}^{T}W(Gu-p)=0\end{array}$$

Here, ${u}_{{\rm{prior}}}$ is the vector of attenuation coefficients of prior image and $\lambda \in [0,1]$ is the weight parameter of prior image constraint terms in the objective function. The first prior image constraint term *R*_*p*_ in Eq. (2) constrains the reconstructed image toward the prior image ${u}_{{\rm{prior}}}$, while the second one *R*_*g*_ works as a global constraint term to overcome the ill-posedness in LDCT. Effect of the two constraints is modulated through the parameter *λ* in Eq. (2).

Nevertheless, two limits exist for the PICCS model in Eq. (2). The first one is the position displacement between a prior image and the current target image. A high quality prior image with exactly matched position correspondence is often not available. The second one is that the unitary analysis transform model (*i.e*. global and prior image constraint are both TV constraint) is used in most PICCS methods. The global constraint *R*_*g*_ and prior image constraint *R*_*p*_ give penalization with respect to the information on anatomical structures and residuals, which inherently have significant morphological discrepancy. The L1 norm based TV constraint is not effective in tackling with the versatile features in practical reconstructions. Some blocky artifacts and smeared details tends to appear in the reconstructed LDCT images for the PICCS reconstruction with TV constraints.

In this study, to overcome these limits, we improve PICCS reconstructions by imposing two discriminative feature dictionary constraints built specifically for the desirable tissue attenuation features and the noise-artifacts residual features in the terms in the PICCS framework. The feature dictionary constraints to solve^29–32^:

3 $${\alpha }^{\ast }=\text{arg}\,\mathop{\min }\limits_{\alpha }\sum _{s}({\Vert {E}_{s}u-D{\alpha }_{s}\Vert }_{2}^{2}+v{\Vert {\alpha }_{s}\Vert }_{0})$$

where *D* is the pre-defined feature dictionary, and *E*_*s*_ denotes an operator to extract the *s*-th 3D patch in the volume space, ${\alpha }_{s}$ is the sparse representation coefficients of ${E}_{s}u$ and *v* is the Lagrange multiplier. Then the corresponding developed DP-PICCS framework is formulized in the following minimization problem:

4 $$\begin{array}{rcl}\{{u}^{\ast },{\alpha }_{r}^{\ast },{\alpha }_{t}^{\ast }\} & = & \mathop{\text{arg}\min }\limits_{u,{\alpha }_{r},{\alpha }_{t}}[\lambda \sum _{s}({\Vert {E}_{s}(u-{u}_{{\rm{prior}}})-{D}^{r}{\alpha }_{{r}_{s}}\Vert }_{2}^{2}+{v}_{r}{\Vert {\alpha }_{{r}_{s}}\Vert }_{0})\\  &  & +(1-\lambda )\sum _{s}({\Vert {E}_{s}u-{D}^{t}{\alpha }_{{t}_{s}}\Vert }_{2}^{2}+{v}_{t}{\Vert {\alpha }_{{t}_{s}}\Vert }_{0})]\\  &  & s.t.\,\,{(Gu-p)}^{T}W(Gu-p)=0\end{array}$$

where *D* ^*r*^ and *D* ^*t*^ are the pre-defined feature dictionaries which composed of atoms featuring the anatomy structures for normal tissue attenuation and the noise-artifacts in the reconstruction image, ${\alpha }_{{r}_{s}}$ and ${\alpha }_{{t}_{s}}$ are the sparse coding, ${v}_{r}$ and ${v}_{t}$ are the Lagrange multiplier. Here, the **discriminative prior (DP)** is reflected in two aspects: the discriminative representation with the dictionaries *D* ^*r*^ and *D* ^*t*^, and the high-quality prior image ${u}_{{\rm{prior}}}$ generated by the discriminative feature representation (DFR) method in^20^.

### Implementation of the DP-PICCS method

#### The prior image construction

In the implementation of the DP-PICCS method, the initial iteration is set to the FDK reconstruction, and then the DFR approach in^20^ is applied as the post-processing method to generate the prior image. The discriminative composite dictionary $D=[{D}^{t},{D}^{r}]$ is composed of a tissue attenuation feature dictionary *D* ^*t*^ and noise-artifacts residual dictionary *D* ^*r*^. It is noted that the atoms in *D* need not to be inter-independent, which can provide over-complete representations of CT image features. All the overlapped patches in the 3D LDCT volume *u* are represented by the linear combination of the atoms in dictionary *D* with the linear coefficient vectors $\alpha ={[{\alpha }_{t},{\alpha }_{r}]}^{T}$ calculated by the orthogonal matching pursuit (OMP) algorithm. After this, we obtain the processed volume (approximate tissue attenuation featured volume ${\tilde{u}}_{t}$) by DFR: ${\tilde{u}}_{t}=\sum _{s}({E}_{s}^{T}({D}^{t}{\alpha }_{{t}_{s}}))/\sum _{s}({E}_{s}^{T}{E}_{s})$ ^20^.

**Figure 1:**
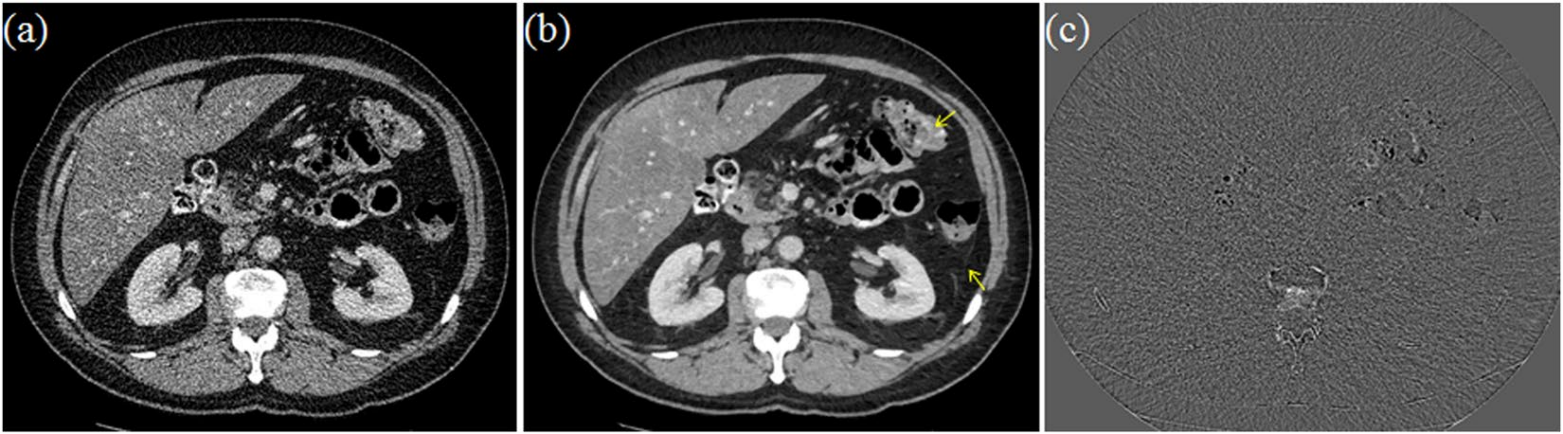
(**a**) LDCT abdomen image; (**b**) Tissue attenuation component image after DFR; (**c**) Residual component after DFR.

Figure 1(a) depicts one slice of clinical LDCT image from one GE Discovery HD750 CT unit. Figure 1(b) and (c) are the corresponding DFR processed image and the noise-artifacts residual component. We can see the DFR approach works well in separating undesirable noise-artifacts features from the original LDCT images, and has the potential to provide a high quality prior image (${u}_{{\rm{prior}}}={\tilde{u}}_{t}$).

#### Iteration reconstruction

With the initial prior image ${u}_{{\rm{prior}}}^{0}$, tissue attenuation feature dictionary *D* ^*t*^ and noise-artifacts residual feature dictionary *D* ^*r*^, Eq. (4) is in fact a non-convex L_0_-norm constrained optimization procedure with respect to *u*. We solve Eq. (4) using an alternative iteratively of image updating task and sparse coding task^33^. Using the FDK reconstruction as the initial volume ${u}^{0}$, the implementation of the DP-PICCS method includes the following three steps:

1. Image update:
  5 $${u}^{k+1}=\text{arg}\,\mathop{\min }\limits_{u}\frac{\beta }{2}{(Gu-p)}^{T}W(Gu-p)+[\lambda \sum _{s}{\Vert {E}_{s}(u-{u}_{{\rm{prior}}}^{n})-{D}^{r}{\alpha }_{{r}_{s}}^{k}\Vert }_{2}^{2}+(1-\lambda )\sum _{s}{\Vert {E}_{s}u-{D}^{t}{\alpha }_{{t}_{s}}^{k}\Vert }_{2}^{2}]$$
  where the superscript *k* and *n* are the update index, *β* is the global parameter for the fidelity term.
2. Tissue attenuation feature sparse coding:
  6 $${\alpha }_{{t}_{s}}^{k+1}=\text{arg}\,\mathop{\min }\limits_{{\alpha }_{{t}_{s}}}\,\,{\Vert {E}_{s}{u}^{k+1}-{D}^{t}{\alpha }_{{t}_{s}}\Vert }_{2}^{2}\,\,\,\,\,\,\,\,\,s.t.\,\,\,\,\,\,\,{\Vert {\alpha }_{{t}_{s}}\Vert }_{0}\le {T}_{t}\,,\,\,\,\,\,\,\,\forall s\,$$
3. Residual feature sparse coding:

7 $${\alpha }_{{r}_{s}}^{k+1}=\text{arg}\,\mathop{\min }\limits_{{\alpha }_{{r}_{s}}}\,\,{\Vert {E}_{s}({u}^{k+1}-{u}_{{\rm{prior}}}^{n})-{D}^{r}{\alpha }_{{r}_{s}}\Vert }_{2}^{2}\,\,\,\,\,\,\,\,\,s.t.\,\,\,\,\,\,\,{\Vert {\alpha }_{{r}_{s}}\Vert }_{0}\le {T}_{r}\,,\,\,\,\,\,\,\,\forall s\,$$

where *T*_*r*_ and *T*_*t*_ are the sparsity level parameters which limiting the maximum atom numbers in the two dictionaries used for a 3D patch sparse coding.

The sup-problem of Eq. (5) is a typical quadratic form. By the separable paraboloid surrogate method, it can be optimized as^28^:

8 $${u}^{k+1}={u}^{k}-\frac{{G}^{T}W(G{u}^{k}-p)+\frac{1}{\beta }{\frac{\partial R(u)}{\partial u}|}_{u={u}^{k}}}{{G}^{T}WGI+\frac{1}{\beta }{\frac{{\partial }^{2}R(u)}{\partial {u}^{2}}|}_{u={u}^{k}}}$$

where $R(u)=\lambda {R}_{p}(u-{u}_{{\rm{prior}}})+(1-\lambda ){R}_{g}(u)=\lambda \sum _{s}{\Vert {E}_{s}(u-{u}_{{\rm{prior}}}^{n})-{D}^{r}{\alpha }_{{r}_{s}}^{k}\Vert }_{2}^{2}+(1-\lambda )\sum _{s}{\Vert {E}_{s}u-{D}^{t}{\alpha }_{{t}_{s}}^{k}\Vert }_{2}^{2}$, *I* is a unity vector. Then, the solution of volume *u* becomes:

9 $${u}^{k+1}={u}^{k}-\frac{{G}^{T}W(G{u}^{k}-p)+\frac{2(1-\lambda )}{\beta }\sum _{s}{E}_{s}^{T}({E}_{s}{u}^{k}-{D}^{t}{\alpha }_{{t}_{s}}^{k})+\frac{2\lambda }{\beta }\sum _{s}{E}_{s}^{T}({E}_{s}({u}^{k}-{u}_{{\rm{prior}}}^{n})-{D}^{r}{\alpha }_{{r}_{s}}^{k})}{{G}^{T}WGI+\frac{2}{\beta }\sum _{s}{E}_{s}^{T}{E}_{s}\,}$$

The sparse coefficients ${\alpha }_{{t}_{s}}^{k+1}$ and ${\alpha }_{{a}_{s}}^{k+1}$ in Eqs (6) and (7) can be solved by the greedy strategy based BP, MP or OMP algorithm^29–32,34^. In this paper, the Batch-OMP algorithm is considered as the solver for improving high computational efficiency^35^.

**Figure 2:**
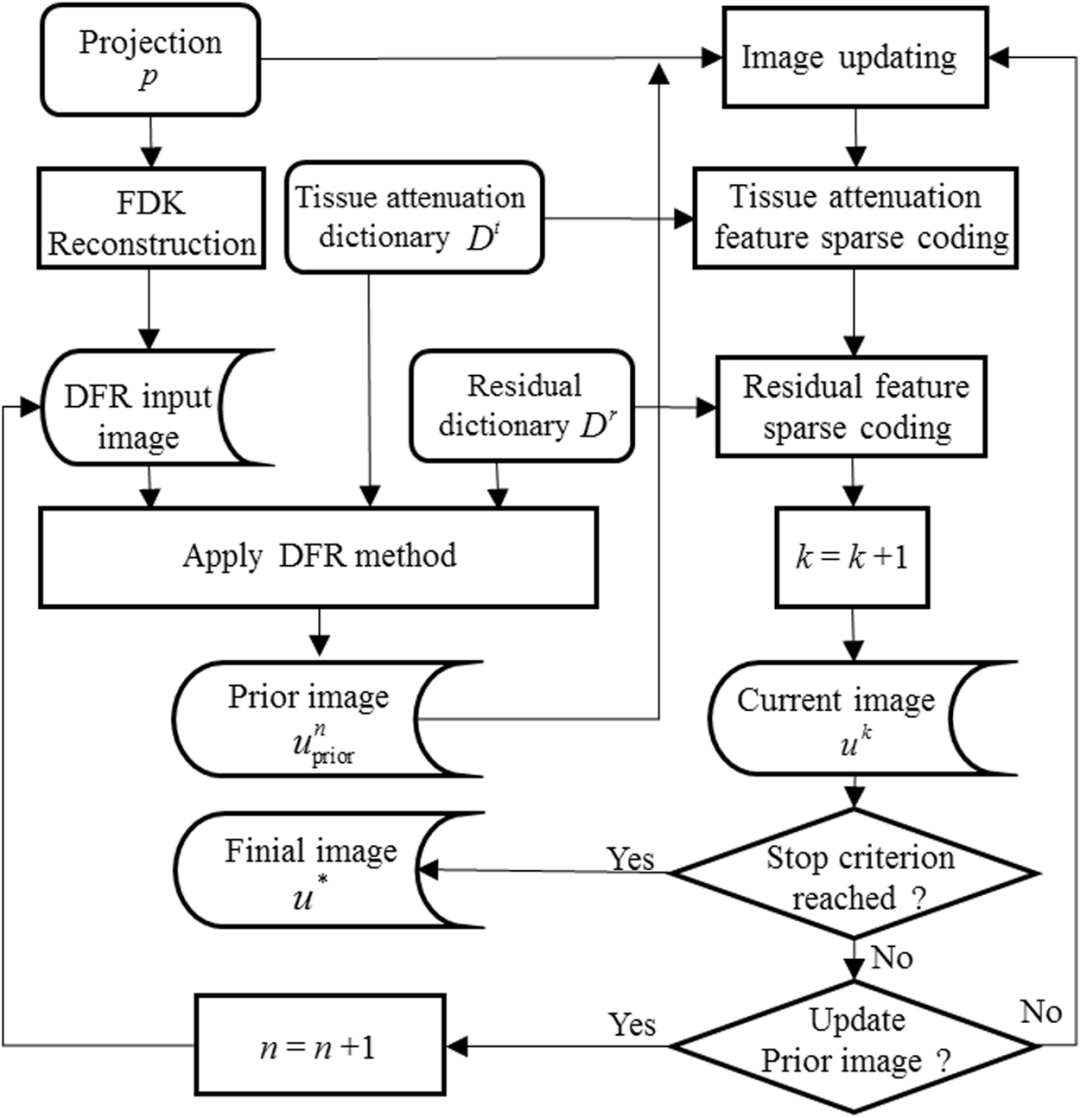
A flowchart of the proposed DP-PICCS algorithm for LDCT reconstruction.

The overall DP-PICCS algorithm is implemented based on **Algorithm 1**, which contains two loops: the inside loop labeled by *n* for prior image update (the total inside iteration number is *N*_max_), and the outside loop labeled by *k*. To get a stable solution, the whole reconstruction proceeds until the image update between two consecutive iterations falls below a pre-defined threshold *θ* (${\Vert {u}^{k+1}-{u}^{k}\Vert }_{2}/{\Vert {u}^{k}\Vert }_{2}\le \theta$). In practice, the threshold parameter *θ* is hard to be normalized for different objective function and the measured projection. So, in this study we just use *K*_max_ and *N*_max_ as the simply stopping iteration numbers for the outer loop and inner loop, respectively. The full flowchart of the proposed DP-PICCS algorithm is shown in Fig. 2 
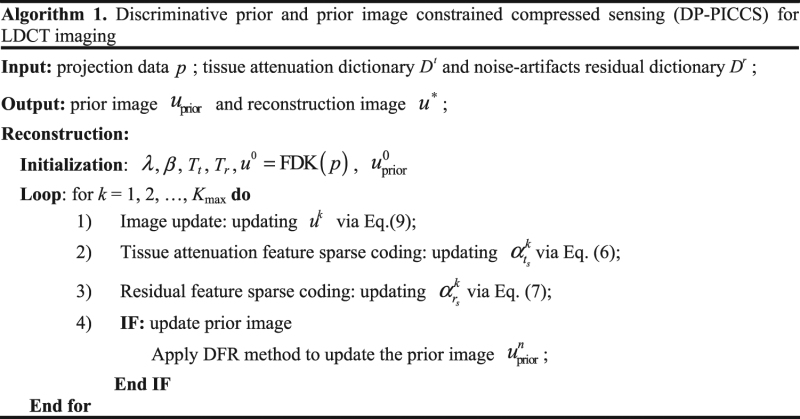

### Construction of the Feature Dictionaries

**Figure 3:**
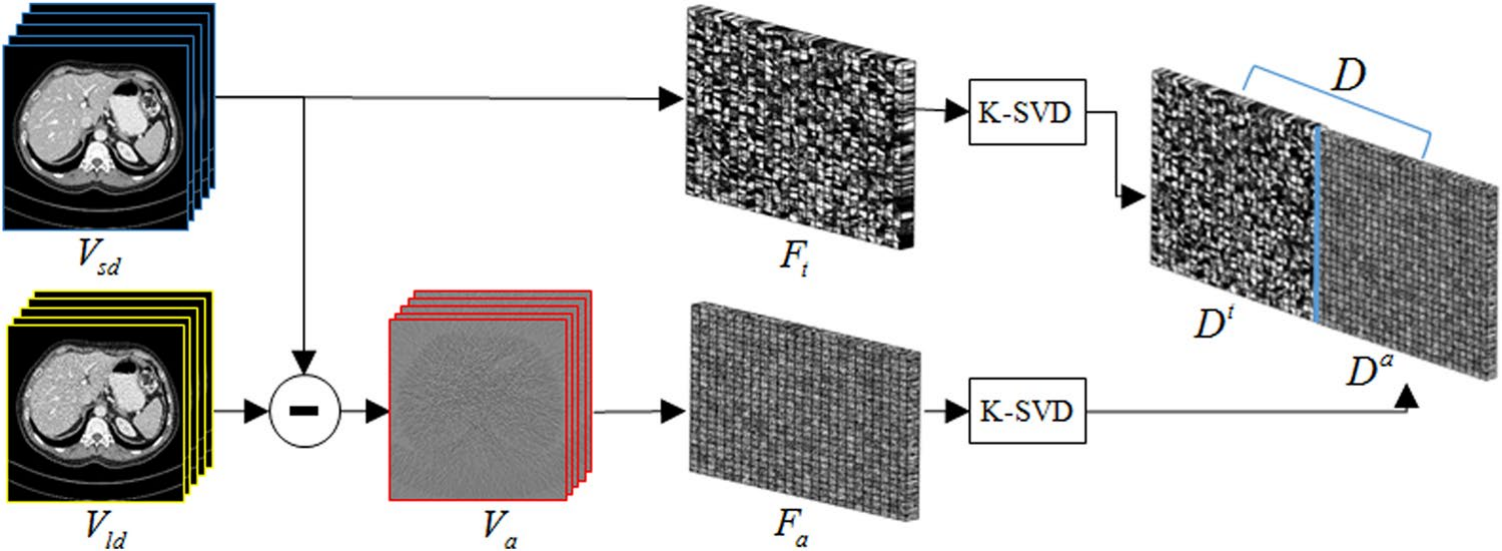
Outline of the feature dictionaries construction.

The DFR approach considers a LDCT volume *u* as the sum of a volume with normal tissue attenuation features and a volume with noise-artifacts residual features^20^. So atoms with distinctly different features should be used to represent the different image constraint terms. With an aim to giving specific sparse representation, we build the two dictionaries using the SDCT projections acquired from the same CT scanner as the target LDCT images to reconstruct, in which the same scan protocol except the tube current is used. Performance of the proposed DP-PICCS method is highly determined by tissue attenuation feature dictionary *D* ^*t*^ and residual feature dictionary *D* ^*r*^, which are respectively the collections of the atoms learned from sample patches. Schemed in Fig. 3, this strategy of dictionary construction includes the following two steps:

***Step 1***, **Sample patches preparation:** From the same CT scanner geometry as the LDCT images to reconstruct, we acquire the SDCT image samples *V*_*sd*_. Then, from this SDCT images, a set of LDCT images *V*_*id*_ are simulated via Eq. (10) and FDK reconstruction with ramp kernel.

10 $${N}_{m}={\rm{Poisson}}\,\{{N}_{0m}\,{e}^{-{p}_{m}}+{r}_{m}\},\,m=1,2\cdots M$$

where *N*_*m*_, *N*_0*m*_ and *r*_*m*_ are the numbers of transmitted photons, incident x-ray photons and read-out noise along the *m-*th X-ray path, respectively. ${P}_{m}$ is the attenuation integral of the *m-*th X-ray path. Note that a lower number of incident photons ${N}_{0m}$ is used to ensure a rich information of residual features in Eq. (10). We then extract tissue attenuation features patches ${F}_{t}$ from the SDCT volume ${V}_{sd}$, and the extract residual patches *F*_*r*_ from the difference volume ${V}_{r}$ between the two matched SDCT and LDCT. To avoid great many training samples for calculation, the extraction operation is performed with a 3D interval Δ_*D*_ specified by the intervals Δ_*x*_, Δ_*y*_, and Δ_*z*_ in the three directions (axes *x*, *y* and *z*).

***Step 2***, **Dictionary construction**: Here, the dictionaries *D* ^*t*^ or *D* ^*r*^ are trained from the ${F}_{t}$ or ${F}_{r}$ by solving the following minimization problem via K-Singular Value Decomposition (K-SVD) method^29–32^:

11 $$\mathop{\min }\limits_{{D}^{t},{{\rm{\Gamma }}}_{t}}{\Vert {F}_{t}-{D}^{t}{{\rm{\Gamma }}}_{t}\Vert }_{2}^{2}\,\,s.t.\,{\Vert {{\rm{\Gamma }}}_{t}\Vert }_{row-0}\le {T}_{L}\,$$

12 $$\mathop{\min }\limits_{{D}^{r},{{\rm{\Gamma }}}_{r}}{\Vert {F}_{r}-{D}^{r}{\Gamma }_{r}\Vert }_{2}^{2}\,\,\,s.t.{\Vert {{\rm{\Gamma }}}_{r}\Vert }_{row-0}\le {T}_{L}$$

where Γ_*t*_ and Γ_*r*_ are the sparse coefficient arrays for each block in *F*_*t*_ and *F*_*r*_, and are alternatively updated with the dictionary atoms in K-SVD algorithm. ${T}_{L}$ is the sparsity level limiting the term ${\Vert .\Vert }_{row-0}$, a pseudo-norm counting the number of non-zero coefficients in each block representation. It should be noted that the atom number of the learned dictionaries is much smaller than the block number in *F*_*t*_ and *F*_*r*_.

## Experiment and Results

To evaluate the performance of the proposed DP-PICCS method, the FDK method (with ramp filter), the DFR post processing method (DFR-post)^20^, the iterative TV minimization based reconstruction^13^, the PICCS method with TV constraint and DFR-post prior image (PICCS_DFR_) and a global feature dictionary-based statistical iterative reconstruction (GDSIR) approach^16^ were adopted for comparison.

The traditional PICCS algorithm under the TV constraint framework and DFR-post prior image (PICCS_DFR_) is formulated as^24^:

13 $$\begin{array}{rcl}{u}^{\ast } & = & \text{arg}\,\mathop{\min }\limits_{u}[\lambda {\Vert {\rm{TV}}(u-{u}_{\mathrm{DFR}-\mathrm{post}})\Vert }_{1}+(1-\lambda ){\Vert {\rm{TV}}(u)\Vert }_{1}]\,\\  &  & s.t.{(Gu-p)}^{T}W(Gu-p)=0\end{array}$$

where the sparsifying transform ${\rm{TV}}(\bullet )$ is the TV constraint^12^.

The GDSIR is a patch-based approach by extracting the prior information via a global dictionary trained from a high-quality CT image, and the associative image reconstruction is equivalent to solve the following minimization problem^25^:

14 $$\begin{array}{rcl}\{{u}^{\ast },{\alpha }^{\ast }\} & = & \text{arg}\,\mathop{\min }\limits_{u,\alpha }\,(\sum _{s}({\Vert {E}_{s}u-{D}^{g}{\alpha }_{s}\Vert }_{2}^{2}+{\nu }_{s}{\Vert {\alpha }_{s}\Vert }_{0})\,)\\  &  & s.t.{(Gu-p)}^{T}W(Gu-p)=0\end{array}$$

In this study, the global dictionary *D* ^*g*^ was the same as tissue attenuation dictionary in DFR (${D}^{g}={D}^{t}$), and an alternating minimization scheme described in^16^ is applied to find the solution of Eq. (14).

**Figure 4:**
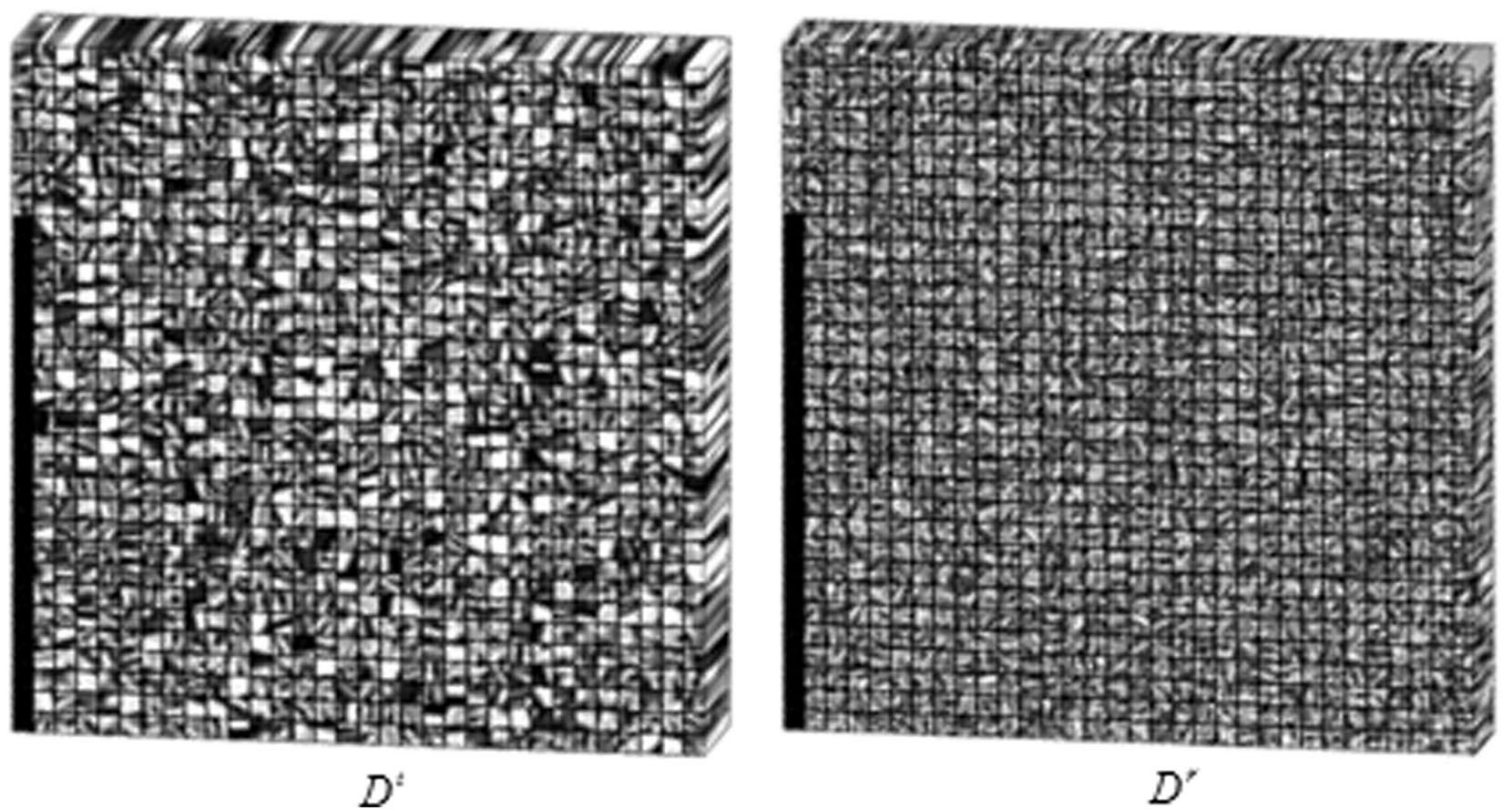
The constructed 3D tissue attenuation feature dictionary and residual feature dictionary for experiments.

All algorithms were implemented in Matlab 8.3 environment on a personal computer (Intel i7-4790k CPU and 32-GB RAM). The reconstruction parameters used for all the experiments are listed in Table 1. All reconstructions were stopped after 50 iterations and updated to a stable solution. Two dictionaries *D* ^*t*^ and *D* ^*r*^ were respectively built using four set clinical abdomen SDCT volumes containing total 100 slices acquired from the Siemens CT scanner following the steps in the Fig. 3. The sparsity level *T*_*L*_ was set 8, the atom number *C* and patch size *B* in the dictionary construction stage were respectively set to 1000 and 8 × 8 × 5. The built 3D feature dictionaries were illustrated in Fig. 4. We can observe that the atoms in the two dictionaries can well reflect the attenuation features and the noise-artifacts features in CT images. It is also noted that some atoms in the tissue attenuation dictionary *D* ^*t*^ present the textures with random intensity distribution, which were actually related to some background regions in the SDCT images when mean values are removed. Though not presenting obvious structural textures, these atoms with random intensity distribution in *D* ^*t*^ practically contribute to the restoration of realistic SDCT image textures.

**Table 1:** Parameter Settings Of Different Iterative Reconstruction Methods.

Dataset	PICCS_DFR_	GDSIR	DP-PICCS
Phantom Case D1	*λ* = 0.4, *β* = 4 × 10^−3^	*λ* = 1.4, *L* = 8, *ε* = 1.6 × 10^−3^	*λ* = 0.36, *β* = 0.24, *T*_*t*_ = 8, *T*_*a*_ = 6
Phantom Case D2	*λ* = 0.3, *β* = 2 × 10^−3^	*λ* = 2.3, *L* = 8, *ε* = 2.6 × 10^−3^	*λ* = 0.35, *β* = 0.21, *T*_*t*_ = 8, *T*_*a*_ = 8
Phantom Case D3	*λ* = 0.3, *β* = 1.4 × 10^−3^	*λ* = 2.3, *L* = 8, *ε* = 3.6 × 10^−3^	*λ* = 0.35, *β* = 0.19, *T*_*t*_ = 8, *T*_*a*_ = 8
Clinical Abdomen Data A and Data B	*λ* = 0.3, *β* = 2.5 × 10^−3^	*λ* = 1.8, *L* = 8, *ε* = 3.6 × 10^−3^	*λ* = 0.35, *β* = 0.2, *T*_*t*_ = 8, *T*_*a*_ = 8

### Anthropomorphic torso phantom simulation study

#### Data Acquisition

**Figure 5:**
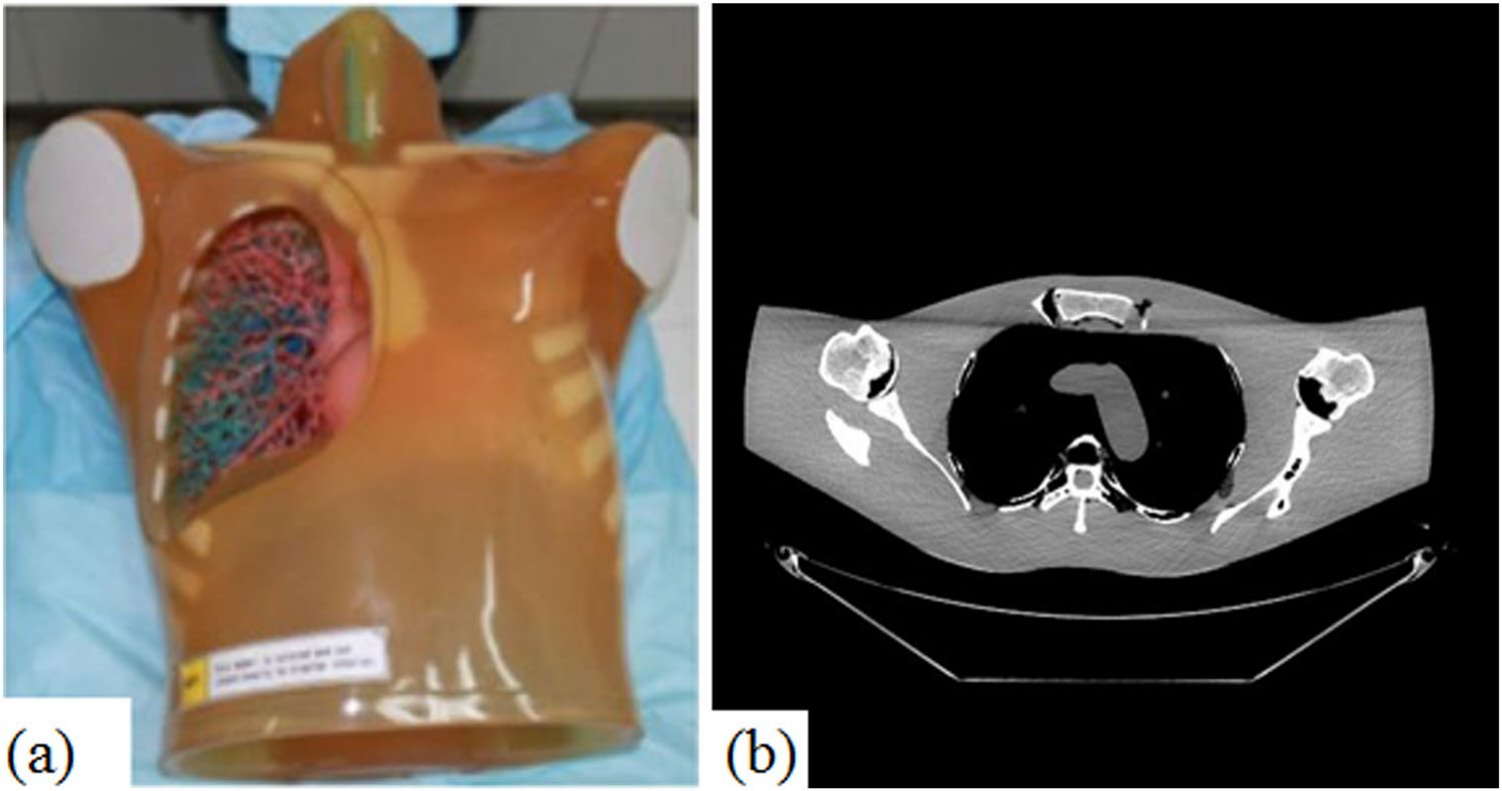
(**a**) The anthropomorphic torso phantom (Kyoto Kagaku co. LTD Japan); (**b**) Middle slice of this torso phantom volume axial view.

In torso phantom simulation study, a set of high quality CT volume (SDCT images) was scanned at 600mAs protocol on a GE Discovery HD750 CT scanner (tube voltage: 120KVp). The high quality torso phantom volume was reconstructed by FDK method with standard ramp filter. The torso phantom and the middle slice of axial views are illustrated in Fig. 5. For simulation, the source-to-axial distance and source-to-detector distance of the trajectory was respectively set to 57.3 cm and 101 cm. The detector panel has 960 × 256 elements with element size 1.024 × 1.10165mm^2^. 360 projections covered one cycle axial scan were uniformly collected. Three different Poisson noise intensity were superimposed onto the raw projection data to synthesize low dose projection data (Photon intensities are ${b}_{m}$ = 5×10^4^, 1 × 10^4^ and 5 × 10^3^ in Eq. (10) for Case D1, Case D2 and Case D3, respectively). For all the iterative algorithms, the whole projections were split into 20 subsets to accelerate computation.

#### Visual Assessment

**Figure 6:**
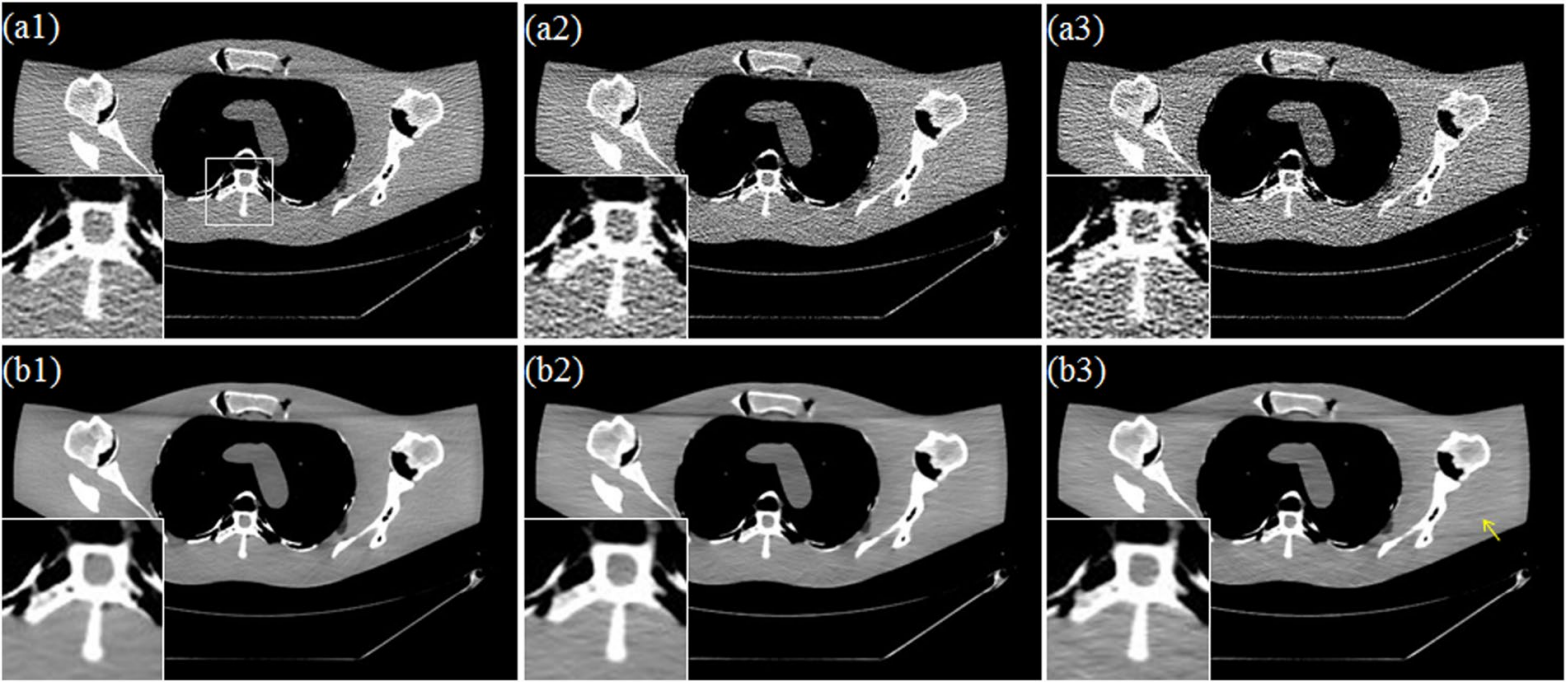
FDK reconstruction images and corresponding DFR-post images. (**a1**–**a3**) are the FDK reconstructed images correspond to Case D1, Case D2 and Case D3, respectively; (**b1**–**b3**) are the DFR-post images correspond to Case D1, Case D2 and Case D3, respectively.

Reconstructions with induced prior image depend heavily on the quality of prior image. From top to bottom, Fig. 6(a1–a3) provides the FDK reconstructed images for the scanner protocols of Case D1, Case D2 and Case D3. Fig. 6(b1–b3) provides the corresponding prior images produced by the DFR-post method form LDCT FDK images. All images are illustrated with the display window center 60HU and display window width 450HU. From the Fig. 6, we can observe the presence of strong noise and artifacts in the FDK reconstruction, which become severer as the doses decrease. From the second row in Fig. 6, the DFR-post method shows good performance in noise-artifacts suppression and structure preservation, and can provide as prior image in good quality and without deformation.

**Figure 7:**
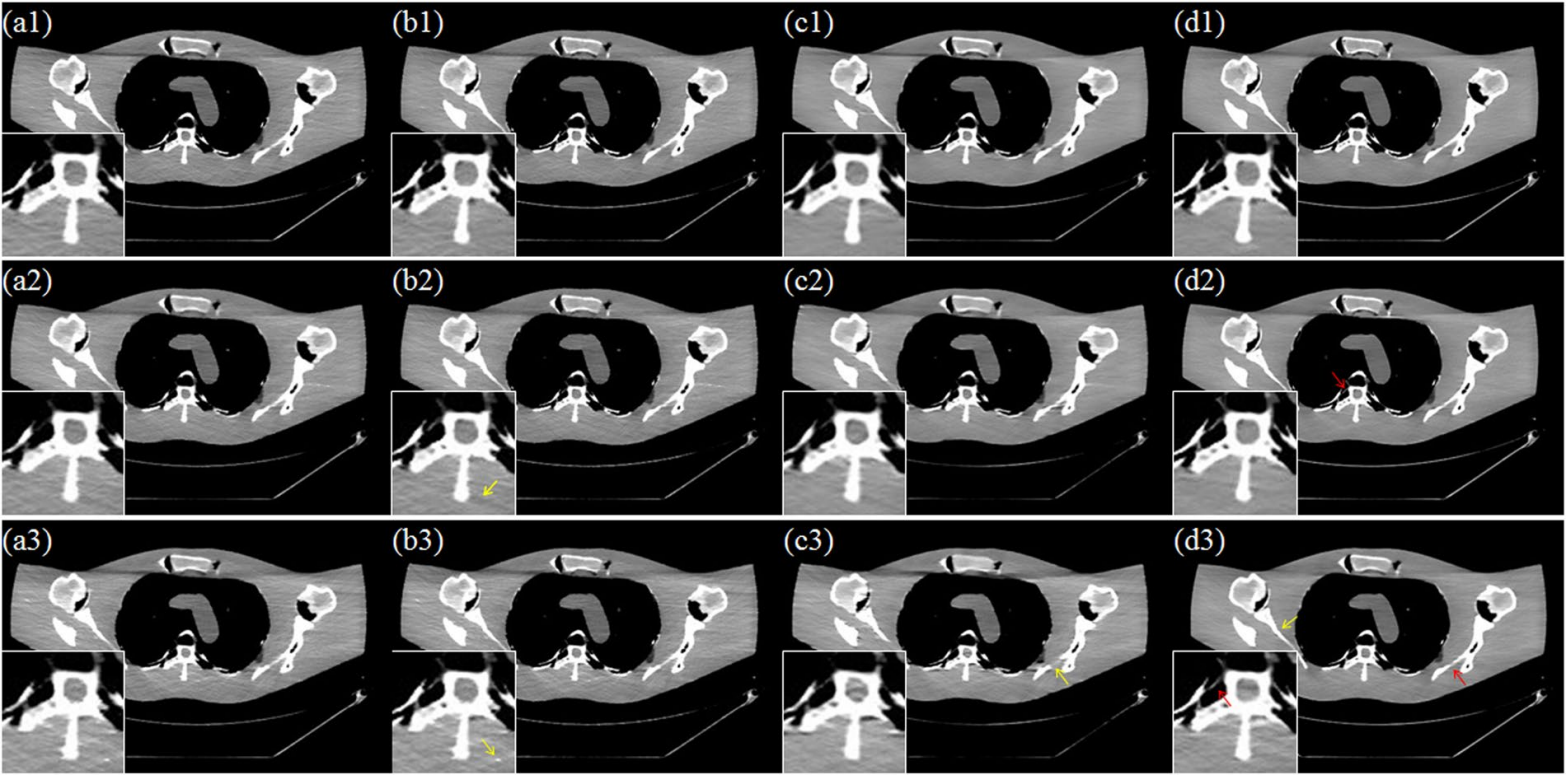
Selected axial views in the simulated phantom reconstruction. From left to right, images reconstructed using the TV, PICCS_DFR_, GDSIR and DP-PICCS methods from Case D1 (the first row), Case D2 (the second row) and Case D3 (the third row) projection, respectively.

Figure 7 depicts the results from different reconstruction algorithms with three scanner protocols. From top to bottom, the scanner protocols are Case D1, Case D2 and Case D3, respectively. From left to right, the reconstruction algorithms are TV, PICCS_DFR_, GDSIR and DP-PICCS, respectively. We can observe that the smooth regions are plagued by some block and sharp artifacts, which may have resulted from the TV piecewise smooth constraint in TV and PICCS_DFR_ methods results in Fig. 7(a3) and (b3). This is because the gradient variation based TV constraint fails to provide good discrimination ability between desirable tissue structure features and streak-artifacts. In the third columns in Fig. 7, the GDSIR method works well in suppressing noise and artifacts but at the cost of smoothing out some organ edges (see the zoomed region and yellow arrow in Fig. 7(c3)). Comparing the results in all the zoomed regions, we can see that the proposed DP-PICCS method achieves the best image quality in structure retention and noise-artifact suppression for all the three dose levels.

#### PSNR and UQI Measure

Quantitative evaluation is performed using two metrics the peak signal to noise ratio (PSNR) and universal quality index (UQI). Here the PSNR and UQI are calculated via Eqs (15) and (16)^36^:

15 $${\rm{PSNR}}(u,{u}_{GS})=10\,{\mathrm{log}}_{10}(\frac{{u}_{\max }^{2}}{\frac{1}{N}\sum _{n=1}^{N}{({u}_{n}-{u}_{GS,n})}^{2}})$$

16 $${\rm{UQI}}(u,{u}_{GS})=\frac{4{\sigma }_{u,{u}_{GS}}{\overline{uu}}_{GS}}{({\sigma }_{u}^{2}+{\sigma }_{{u}_{GS}}^{2})[{(\bar{u})}^{2}+{({\bar{u}}_{GS})}^{2}]}$$

where *u* represents the reconstructed image from the LDCT, and ${u}_{GS}$ denotes a golden standard image, *n* indicates the voxel index and *N* is the number of voxels, $\bar{u}$ and ${\bar{u}}_{GS}$ are the mean pixel values of *u* and ${u}_{GS}$, respectively, ${\sigma }_{u}$ and ${\sigma }_{{u}_{GS}}$ are the standard deviation of image pixel values of *u* and ${u}_{GS}$, respectively, and ${\sigma }_{u,{u}_{GS}}$ is the correlation coefficient between *u* and ${u}_{GS}$. The UQI is unitless and has a dynamic range of [−1, 1] reflecting the similarity degree between the reconstructed and golden standard images.

**Figure 8:**
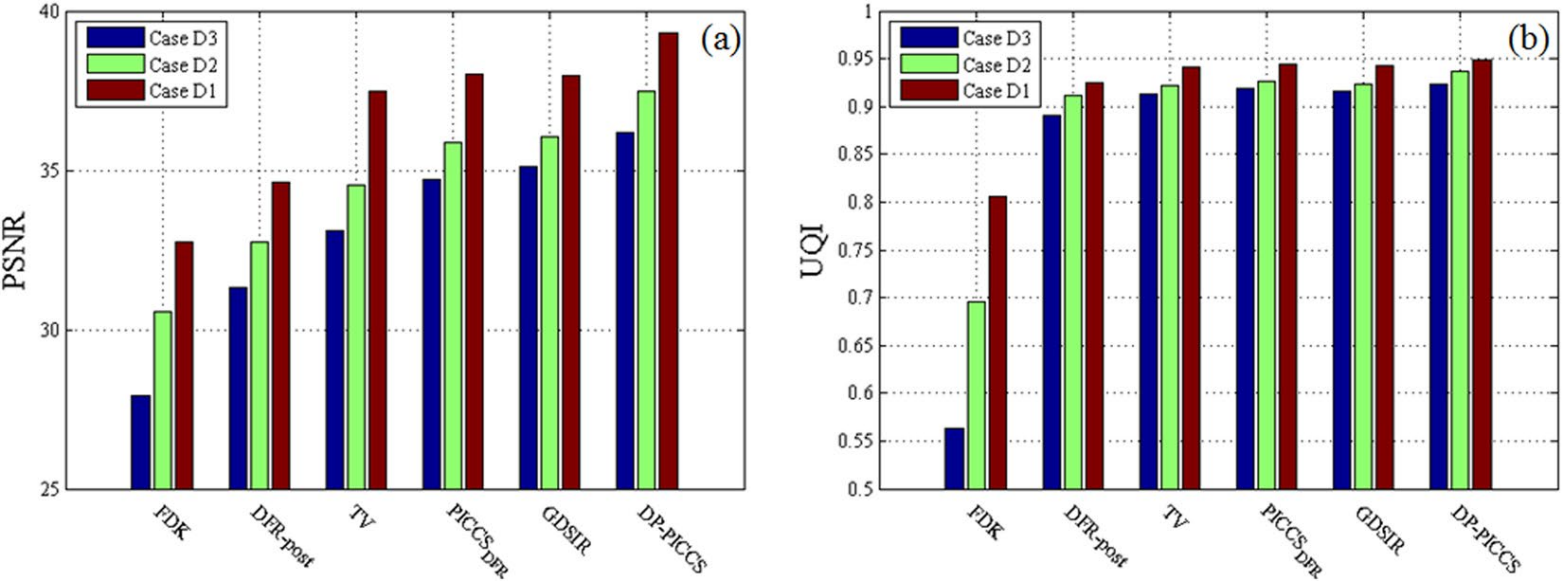
PSNR and UQI measures on the different reconstruction images in Fig. 7. (**a**) PSNR; (**b**) UQI.

In calculating the PSNR and UQI of the reconstructed images, FDK reconstructed SDCT images (tube voltage: 120KVp, tube current: 600mAs, Fig. 5(b)) are used as the golden standard reference. The PSNR and UQI results are shown in Fig. 8. It can be seen in Fig. 8 that the FDK method obtains the worst scores for all the three cases dose levels, and the proposed method performs better than the competing methods in terms of the two metrics (with margins of 1–2 dB for PSNR and 0.01–0.02 for UQI). We can also see that such quantitative results are consistent with the visual performance in Fig. 7. After the LDCT FDK images processed by DFR-post method, the images quality gets significant improvement in both PSNR and UQI. As shown in Fig. 8, the PICCS_DFR_ reconstructed CT images obtained higher quality scores than the TV reconstructed CT images, and such improvement is brought by the use of high quality prior images from the DFR post-processing. The PSNR indexes of GDSIR reconstructed images are higher than those of the TV and PICCS_DFR_ reconstructed images, while the UQI indexes remain in the similar level.

#### Convergence Analysis of the DP-PICCS Method

As described in section II.B, we solve the DP-PICCS reconstruction using split alternating optimization iteration between feature sparse coding and reconstruction image update. In image updates step, sub-problem Eq. (5) is a quadric constraint minimization problem and we solved it with a separable paraboloid surrogate method. This step can be considering a convergence process.

**Figure 9:**
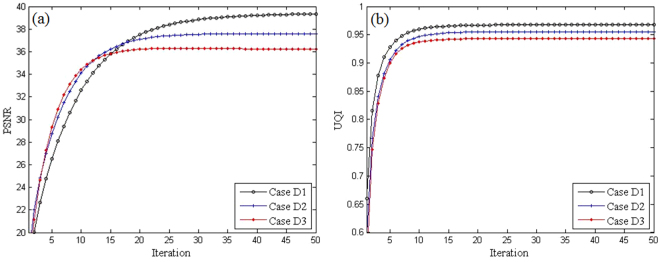
Plots of PSNR and UQI versus DP-PICCS iteration number in Case D1, Case D2 and Case D3. (**a**) PSNR; (**b**) UQI.

In feature sparse coding step, the non-convex sub-problems of sparse coding in Eq. (6) and Eq. (7) are NP-hard problem. To solve them, the Batch-OMP algorithm employs greedy strategy only to obtain a local minimizer in most cases^30,35^. Such alternating process cannot be guaranteed to converge to a global minimum due to the non-convexity L0-norm of the objective function in Eq. (4). To analyze the iteration stability of the DP-PICCS method, the global PSNR and UQI measures on the entire to-be-reconstructed anthropomorphic torso phantom image were calculated. The plotted PSNR and UQI values in Fig. 9 show that the reconstruction quality increases as the iteration proceeds for the proposed algorithm. This observation is confirmed by the study on both torso phantom and clinical abdomen data. Considering the formulation of our problem is similar to that in DLMRI, we can make a similar statement of a stable iteration as in^37^.

### Clinical abdomen data study

#### Data Acquisition

In the clinical abdomen imaging study, two sets of clinical abdomen projection data (Data C1 and Data C2) were provided by the Mayo Clinic (USA), which scanned from a Somatom Definition AS + CT scanner in a helical mode (tube voltage: 100KVp; tube current: 360mAs)^38^. The protocol of this study (data collection and processing) was approved by the institutional ethical review board of the Mayo Clinic and was conducted in accordance with the principles of the 1964 Declaration of Helsinki. Two patients were involved in the experiments. All these patients have given their written informed consent to the participation. A non-conflict of interest for this work was declared. The proposed method was carried out in accordance with the approved guidelines. The data were analyzed fully anonymously. Specific Poisson noise was superimposed onto the raw projection data for each case in the library to synthesize a low dose level that corresponded to 1/4 the standard dose assuming 85mAs for tube current value^38^. The detector has 736 × 64 elements with element size 1.2856 × 1.0947 mm^2^. The source-to-axial distance and source-to-detector distance of the trajectory was respectively set to 59.5 cm and 108.56 cm. The helical trajectory of source was covered by 1152 view angles per cycle with a pitch equal to 0.6. For all the iterative algorithms, the whole projections were split into 32 subsets to accelerate computation.

#### Visual Assessment

**Figure 10:**
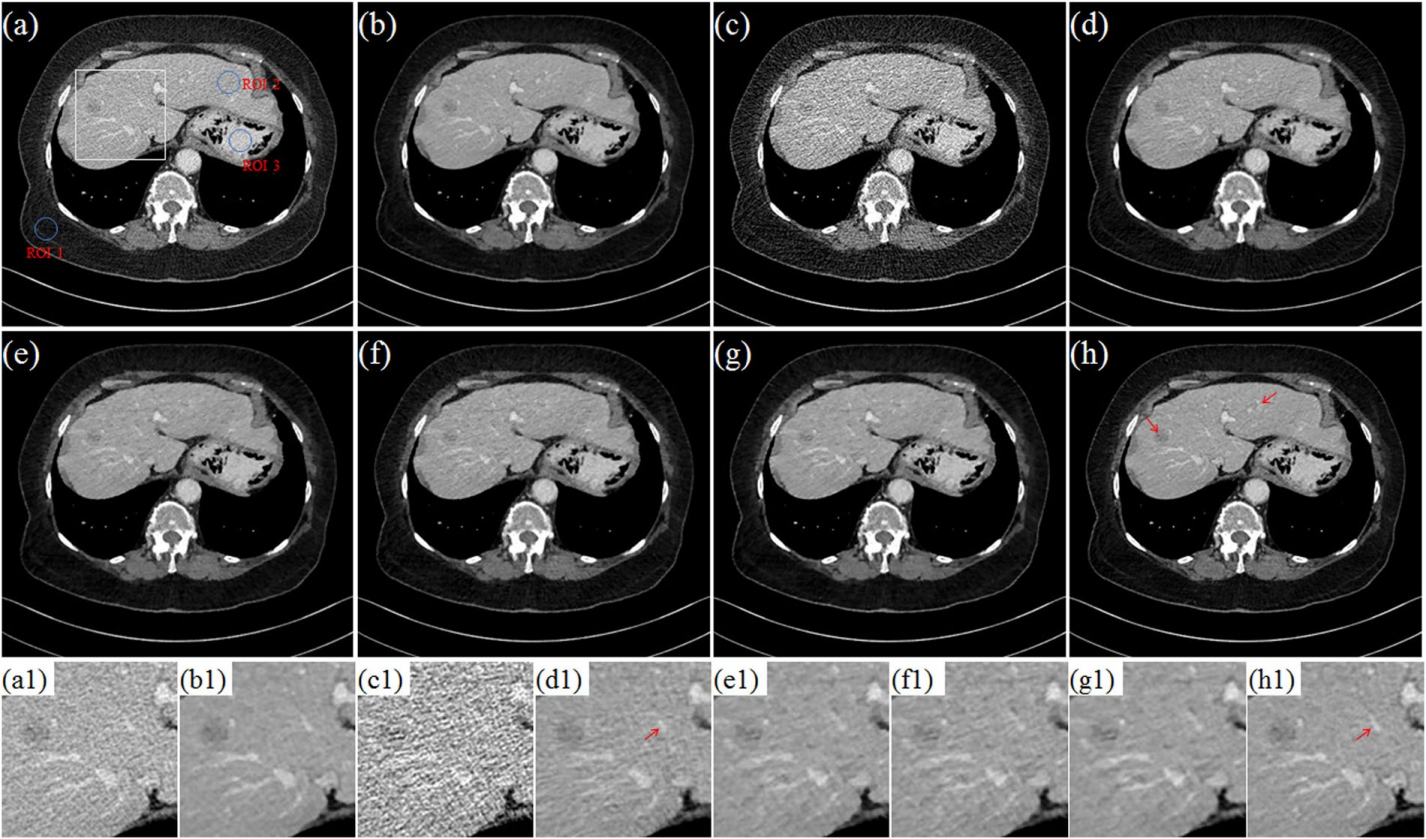
The axial views for Data C1. (**a,b**) are the images reconstructed by the FDK and TV methods for standard dose protocol; (**c–h**) are the images reconstructed by the FDK, DFR-post, TV, PICCS_DFR_, GDSIR, DP-PICCS methods for low dose protocol; (**a1–h1**) are the white zoomed regions of the rectangles delineated in (**a–h**).

**Figure 11:**
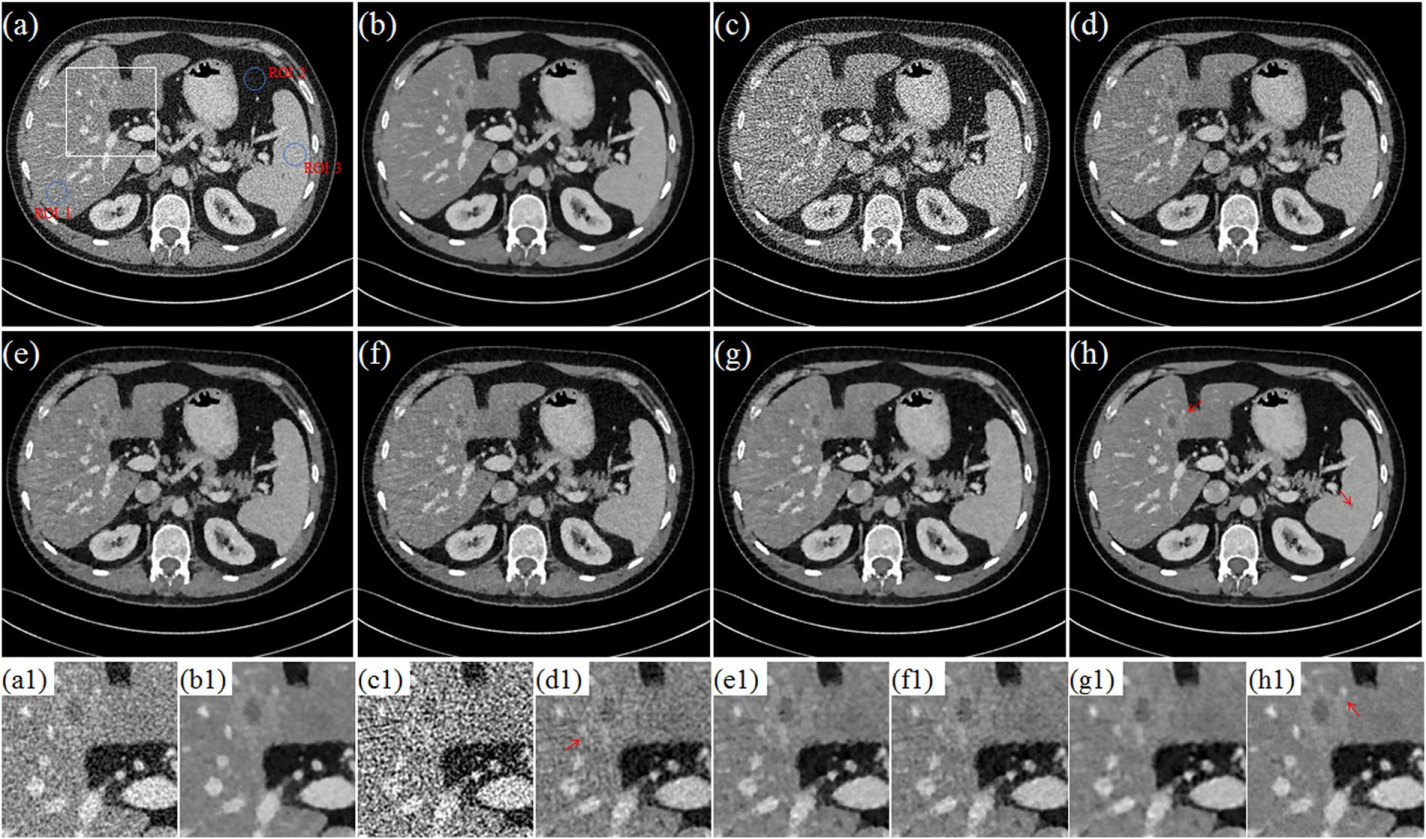
The axial views for Data C2. (**a–b**) are the images reconstructed by the FDK and TV methods for standard dose protocol; (**c–h**) are the images reconstructed by the FDK, DFR-post, TV, PICCS_DFR_, GDSIR, DP-PICCS methods for low dose protocol; (**a1–h1**) are the white zoomed regions of the rectangles delineated in (**a–h**).

Figure 10 and Fig. 11 display the selected axial views from the reconstructed results of Data C1 and Data C2. In Figs 10–11, images (a)–hepatic vein (see the zoomed region illustration(b) are respectively the illustrations for FDK and TV methods for the standard dose protocol, which the TV reconstructed images (Figs 10(b) and 11(b)) are used as reference; images (c)–(h) are respectively the illustrations for FDK, DFR-post, TV, PICCS_DFR_, GDSIR, DP-PICCS methods for the low dose protocol.

From the Figs 10 and 11, we can see that the DFR method works well with effective noise and artifacts suppression in images (b). It is found in Fig. 11(e,f) that the PICCS_DFR_ approach leads to reconstruction in similar quality as the TV algorithm. Though demonstrating good performance in artifacts and noise suppression, the GDSIR method suffers from edge structure blurring of the hepatic vein (see the zoomed region illustration in Figs 10(g1) and 11(g1)). The illustrations in Figs 10 and 11 also show that, though still suffer less from noise-artifacts residual, the DP-PICCS reconstructed LDCT images present a better tiny structure identification than competing methods (see the red arrows in Figs 10 and 11).

**Figure 12:**
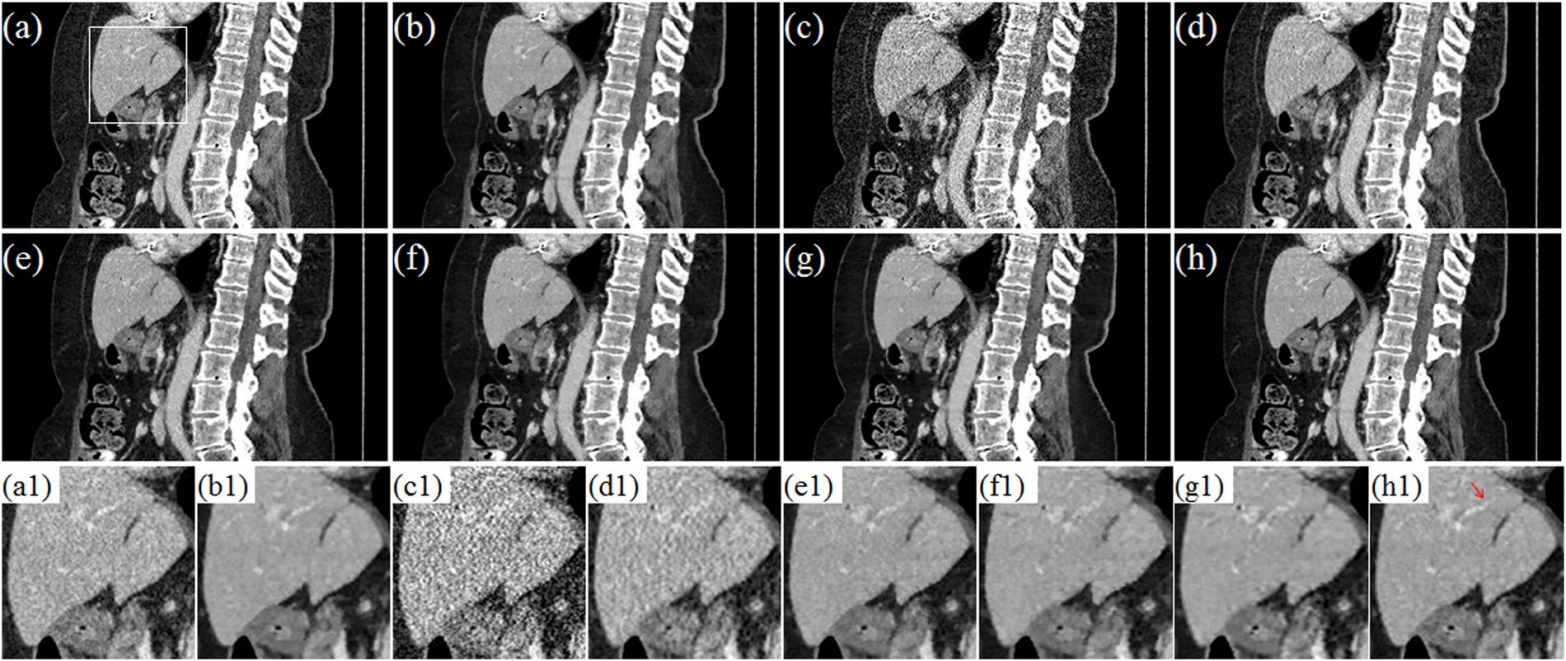
The sagittal views for Data C1. (**a,b**) are the images reconstructed by the FDK and TV methods for standard dose protocol; (**c–h**) are the images reconstructed by the FDK, DFR-post, TV, PICCS_DFR_, GDSIR, DP-PICCS methods for low dose protocol; (**a1–h1**) are the white zoomed regions of the rectangles delineated in (**a–h**).

**Figure 13:**
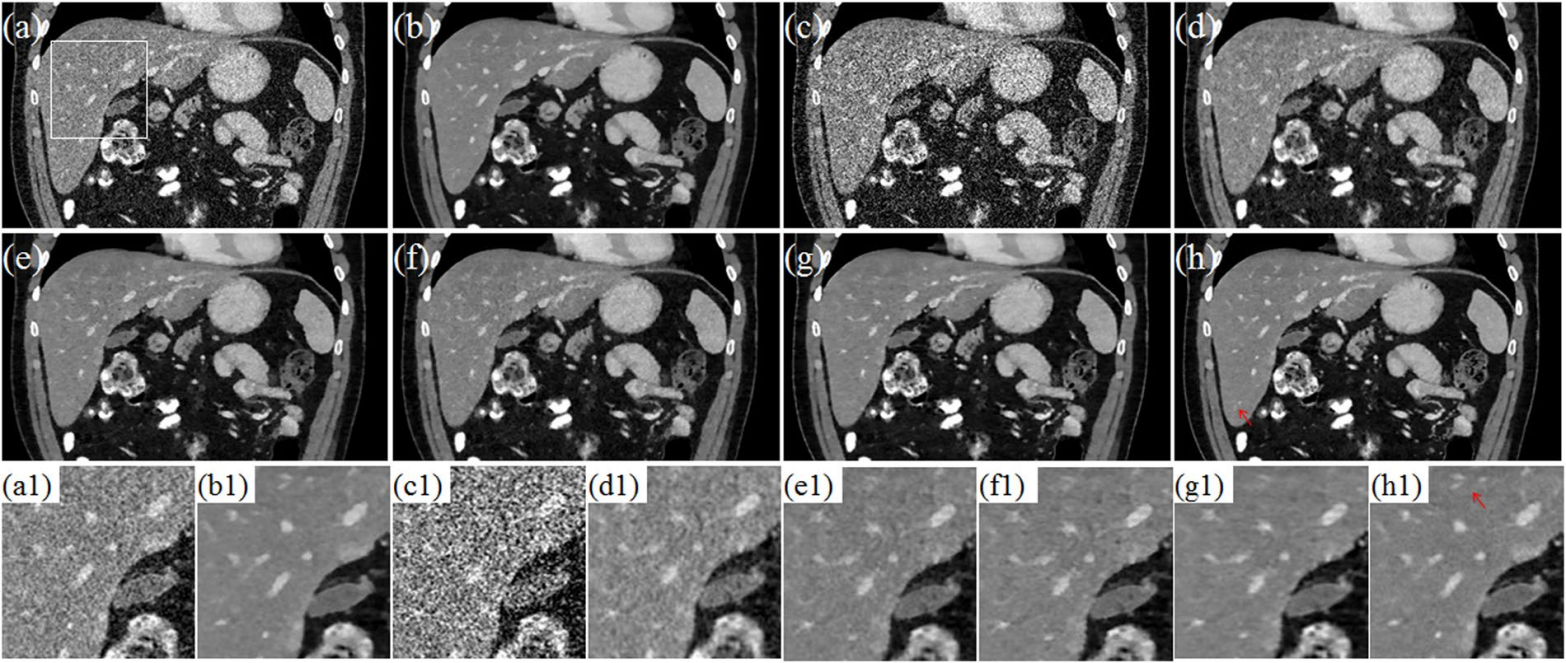
The coronary views for Data C2. (**a–b**) are the images reconstructed by the FDK and TV methods for standard dose protocol; (**c–h**) are the images reconstructed by the FDK, DFR-post, TV, PICCS_DFR_, GDSIR, DP-PICCS methods for low dose protocol; (**a1–h1**) are the white zoomed regions of the rectangles delineated in (**a–h**).

Figures 12 and 13 display the selected sagittal and coronal views from the reconstructed CT volumes of Data C1 and Data C2. In Figs 12 and 13, it can be seen that the proposed DP-PICCS method performs better than the PICCS_DFR_ and GDSIR methods, providing images with improved visual quality if using the TV reconstructed SDCT images (Figs 12(b) and 13(b)) as the references. Compared to other methods, the DP-PICCS method achieves a better preservation of anatomical features (see the zoomed region on liver tissue boundaries and arteries in Fig. 12(h1)) than the TV and PICCS_DFR_ methods. From the Figs 12 and 13(g), we can see that the featured atoms in GDSIR method can be used to provide improved structure preservation in reconstruction. Also, it is found in Figs 12 and 13 that the proposed DP-PICCS method achieves the best performance in noise-artifacts suppression and anatomical features preservation among all competing reconstruction methods (see the structures pointed by red arrows in the Figs 12(h1) and 13(h1)).

#### lSNR and CNR Based Quantification

For clinical reconstruction data quantitative study, two metrics were utilized to give evaluation, namely, local Signal to Noise Ratio (lSNR) and Contrast-to-Noise Ratio (CNR). Here the lSNR and CNR are calculated by Eqs (17) and (18):

17 $${\rm{lSNR}}(u)=\frac{1/N{\sum }_{n=1}^{N}{u}_{n}}{\sqrt{1/N{\sum }_{n=1}^{N}{({u}_{n}-1/N{\sum }_{n=1}^{N}{u}_{n})}^{2}}}$$

18 $${\rm{CNR}}(u,{u}_{BG})=\frac{|\bar{u}-{\bar{u}}_{BG}|}{\sqrt{{\sigma }_{u}^{2}-{\sigma }_{{u}_{BG}}^{2}}}$$

where $\bar{u}$ and ${\bar{u}}_{BG}$ are the mean intensities of the region of interest (ROI) and background region, respectively. ${\sigma }_{u}$ and ${\sigma }_{{u}_{BG}}$ are the associated standard deviations, respectively.

The lSNR values of the three different ROIs in Table 2, as indicated by the blue circles in Figs 10(a) and 11(a), were measured in the Data C1 and Data C2. The methods, *i.e*. DFR-post, TV, PICCS_DFR_, GDSIR, and DP-PICCS; yielded some gains on lSNR over the FDK reconstructions in the three different ROIs. It shows that, for the low-dose case, the FDK method obtains the worst value, and the DP-PICCS method yields the highest value (in metric of lSNR for most ROIs) which are closer to the scores of TV SDCT reconstruction images than competing methods. We can also see that such quantitative results are consistent with the visual comparison in Figs 10-11.

**Table 2:** The lSNR (Unit: dB) Values Of The Clinical Data Results.

Scanner Protocol	Reconstruction Methods	Data C1			Data C2		
		ROI 1	ROI 2	ROI 3	ROI 1	ROI 2	ROI 3
SDCT	FDK	50.15	49.23	54.89	25.43	33.87	38.79
	TV	**90.69**	**114.49**	**109.95**	**111.52**	**118.27**	**126.54**
LDCT	FDK	24.22	21.94	28.47	13.77	15.60	16.78
	DFR-post	72.88	77.63	85.91	42.23	49.36	53.19
	TV	78.73	91.01	101.11	66.28	99.52	93.49
	PICCS_DFR_	75.53	105.05	97.41	94.82	109.31	**136.73**
	GDSIR	79.45	99.93	98.29	87.91	94.34	103.54
	DP-PICCS	**87.86**	**105.73**	**104.59**	**95.72**	**121.51**	114.23

**Figure 14:**
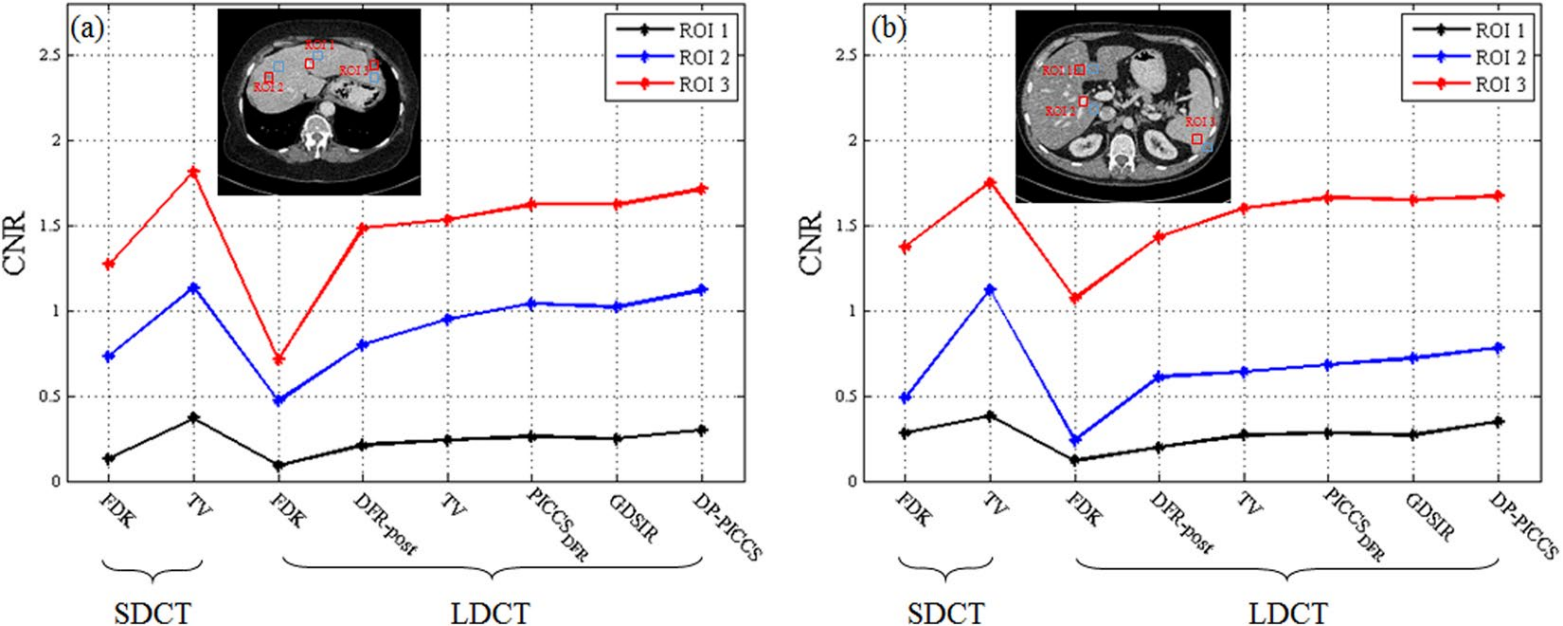
Calculated CNR values on the three ROIs in the two cases of (**a**) Data C1; and (**b**) Data C2.

Figure 14 lists the CNR values of the images reconstructed by the different cases for Data C1 and Data C2. The ROIs for the CNR measure are marked in Fig. 14(a) and (b) (red squares are ROIs and blue squares for backgrounds). It is also observed in Fig. 14 that the DFR-post processing significantly enhances the CNRs of LDCT images (most of them even higher than FDK reconstructed SDCT images) for Data C1 and Data C2, and the DP-PICCS algorithm leads to further higher CNRs. The plots in Fig. 14 also show that most of the CNRs of DP-PICCS indices are nearer to the TV reconstruction in SDCT case. This confirms the above visual comparison that the proposed method has good performance in terms of contrast preservation.

### Sensitivity of dictionary building parameters

**Figure 15:**
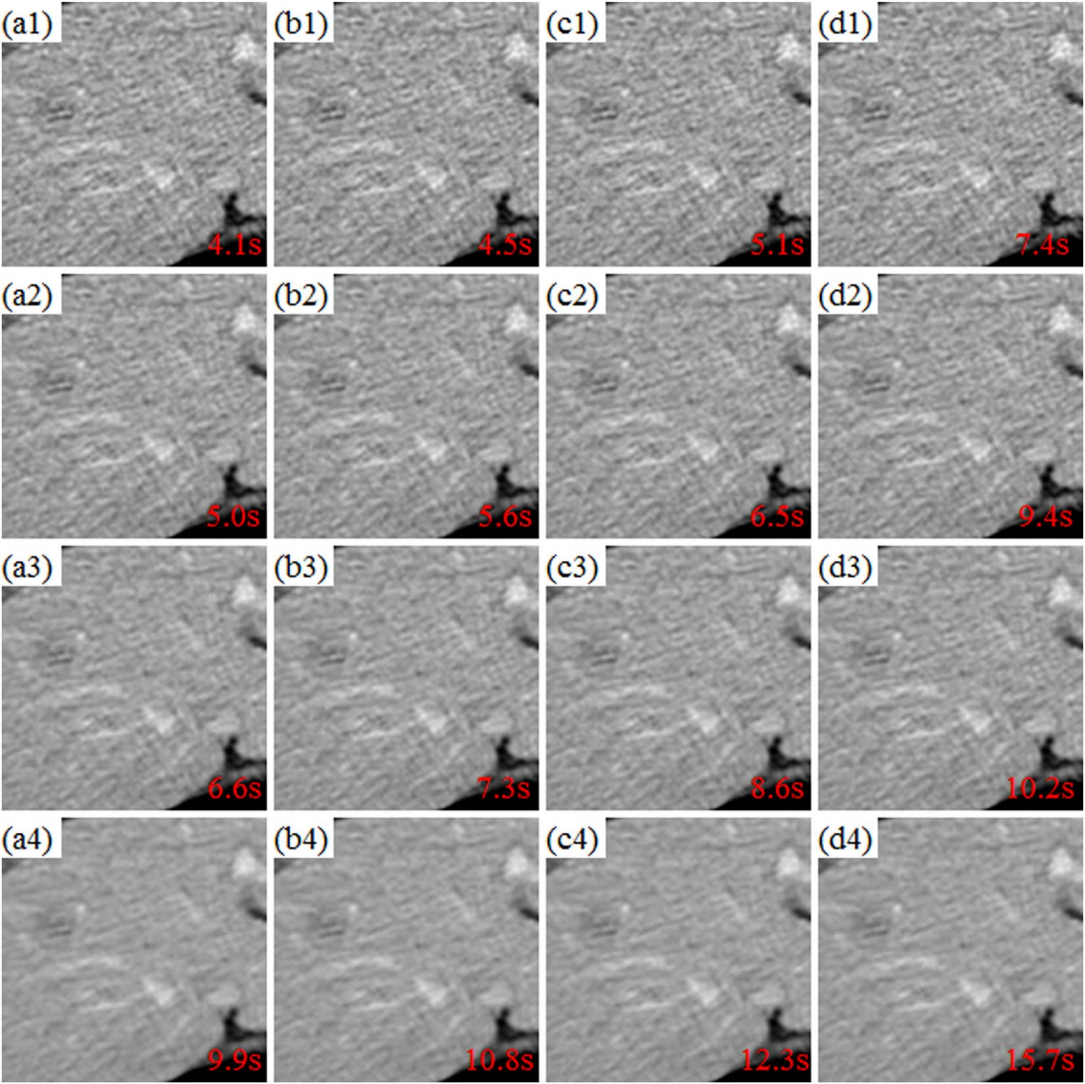
Axial ROI illustrations of the DFR-post prior image when using dictionaries with different atom number and patch size. The rows from above to bottom correspond to the cases with patch sizes *B* = {6 × 6 × 3, 7 × 7 × 4, 8 × 8 × 5, 10 × 10 × 6}, respectively. The columns from left to right correspond to the cases with dictionaries in atom number (500, 500), (1000, 1000), (1500, 1500), (2000, 2000), respectively. The computation cost was tagged in unit second (s) in the right-bottom corner in each ROIs.

Performance of the proposed DP-PICCS is closely related to the parameters in dictionary construction, *e.g*. atom number *C* (dictionary size), and patch size *B*. Figure 15 displays the selected ROI (the LDCT images in Fig. 10(a) processed by DFR-post method) using different atom number *C* and patch size *B*. The computation cost (in unit second (s)) is tagged in the right-bottom corner in each ROI. The rows from above to bottom correspond to the results with *B* = {6 × 6 × 3, 7 × 7 × 4, 8 × 8 × 5, 10 × 10 ×6 }, and the column from left to right respectively correspond to the results related to dictionaries with different atom numbers (500, 500), (1000, 1000), (1500, 1500), (2000, 2000) (in the format of (*C*_t_, *C*_r_) for the dictionaries *D* ^*t*^ and *D* ^*r*^).

We can observe in Fig. 15 that an effective restoration of desirable attenuation information can be obtained if an (1000, 1000) sized dictionary with 8 × 8 × 5 sized atoms are used in patch representation. It is also found in Fig. 15 that almost no visual difference can be discerned when the patch size and dictionary size increase over 8 × 8 × 5 and (1000, 1000). Considering significantly larger computation cost is to be induced when enlarging the dictionary or atoms therein, in this study we use the dictionary size *C*_t_ = *C*_r_ = 1000 and *B* = 8 × 8 × 5 to maintain a good balance of representation accuracy and computation cost.

### Analysis of the parameters in the DP-PICCS reconstruction

Several parameters need to be suitably set in the proposed DP-PICCS method, and these parameters include the fidelity parameter *β*, prior-image constraint parameter *λ* and the sparsity parameters *T*_*t*_ and *T*_*r*_. In general, the fidelity parameter *β* should be well set to balance the data fidelity and prior information constraint terms. The data fidelity term *β* reflected the distance between the iterated images to the measured projection data, so should be lowered in the case of low dose noise projections^17,22^. The parameter *λ* for prior image term should be set according to the prior image quality. In fearture patch sparse coding, the sparsity level parameters *T*_*t*_ and *T*_*r*_ are the maximum numbers of atom permitted in representing each patch. Generally, larger *T*_*t*_ and *T*_*r*_ will give rise to a higher accuracy but increased computational cost. They should be set according to the measured projection noise level in reconstruction.

**Figure 16:**
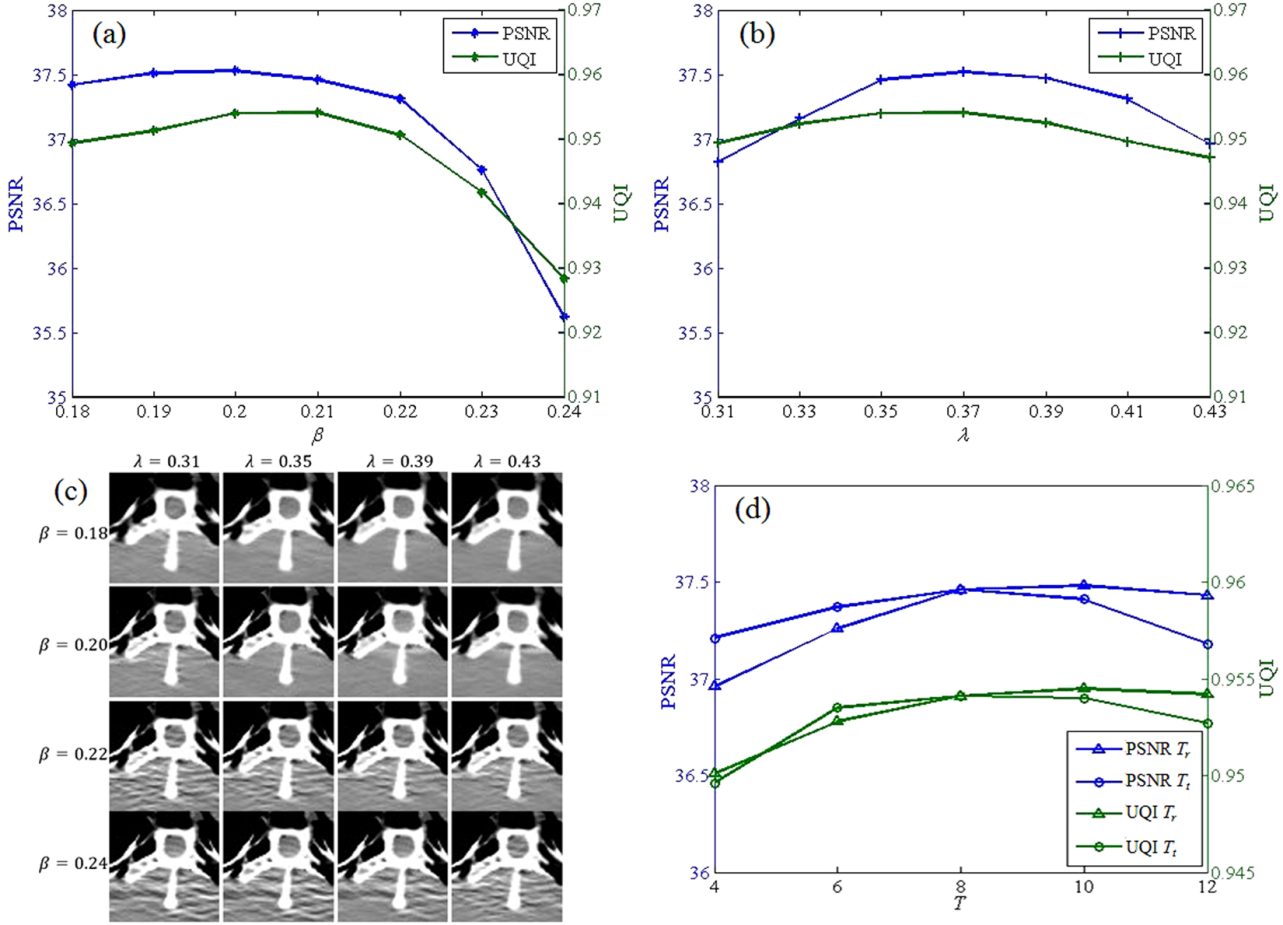
Evaluation of different parameter for torso phantom Case D2 data. (**a**) PSNR and UQI plots versus fidelity parameter *β*; (**b**) PSNR and UQI plots versus prior image parameter *λ*; (**c**) Axial ROI illustrations of reconstructed when using different *β* and *λ*; (**d**) PSNR and UQI plots versus sparsity level *T*_*t*_ and *T*_*r*_. Here, the parameters were analyzed with the other parameters fixed to the values given in Table 1.

Figure 16(a) plots the PSNR and UQI values for different fidelity parameter *β*, using torso phantom Case D2 data with $\lambda =0.35$ and *T*_*t* =_ *T*_*r* = _8. We can observe that the PSNR and UQI values are major affected by the fidelity parameter *β* and attain the highest values when *β* lies around 0.2. Figure 16(b) shows that prior image constraint parameter also have large impacts the reconstruction performance when *λ* ranges from 0.31 to 0.43, and the highest PSNR and UQI value are reached when the *λ* is 0.37. From the plots in Fig. 16(a,b) we can see that the performance of proposed method is quite sensitive to the fidelity parameter *β* and prior image constraint parameter *λ*. The figures in Fig. 16(c) reflects that a large *β* implies an increased fidelity weight of the measured noisy projections which results in increased noise and artifacts in the reconstruction, and a small *β* leads to relatively increased weight of the regularization term which is related to increased smoothing effect. Figure 16(c) also shows that the reconstruction gets smoother when the value of *λ* is increased. Figure 16(d) depicts the PSNR and UQI values for different sparsity level *T*_*t*_ and *T*_*r*_ with the other parameters set based on Table 1. The plots show that the proposed method is robust to sparsity level *T*_*t*_ and *T*_*r*_ from 8 to 12. The PSNR variation is less than 0.5 dB and UQI variation is less 0.01. The reason is due to fact that the same size dictionary is used and eight atoms to twelve atoms are enough to representation these volume patches. So, by taking into consideration both the reconstruction performance and the computation burden, we selected sparsity level *T* *t* = *T* *r* = 8 for the used dictionaries with 1000 atoms in this study.

We select parameters in the proposed approach following the assumption that the same parameters set can be used in reconstructing the data from the same type of CT scanner. Before large volume data reconstruction in our study, the feature dictionaries and parameters are trained to give satisfying results using a small amount of data in the same type.

### Computation cost

Table 3. lists the computation overheads required per iteration (in seconds) for all reconstruction methods considered in our experiments. Both the GDSIR method and the proposed DP-PICCS method suffers from high computational operations to the sparse coding. In our experiments, it takes about 2148 s to train two 3D feature dictionaries (320 × 1000) from 1105142 sample patches for the proposed method. Specifically, we can notice that the GDSIR method and the proposed method require about 63 ± 2 seconds and 154 ± 3 seconds per iteration to reconstruct a 512 × 512 × 30 CT volume in torso phantom data experiment, respectively. In the clinical abdomen data experiment, one DP-PICCS iteration takes about 167 ± 5 seconds to reconstruct a 512 × 512 × 30 CT volume. We can see that the DP-PICCS method is about twice time consuming as much the GDSIR method due to the two 3D feature sparse coding for each volume patch. Besides, one of the biggest advantages of the TV method and PICCS_DFR_ is its low computation complexity.

**Table 3:** Computation Cost Between The DFR-post TV PICCS_DFR_, GDSIR And DP-PICCS Methods (Unit: Second per Iteration).

Method	Phantom data (Size:512 × 512 × 30)	Clinical data (Size:512 × 512 × 30)
DFR-post	219 ± 2	225 ± 3
TV	3 ± 1	5 ± 1
PICCS_DFR_	7 ± 1	11 ± 1
GDSIR	63 ± 2	77 ± 2
DP-PICCS	154 ± 2	167 ± 5

## Conclusion

The proposed DP-PICCS method is an extension of the PICCS method by using prior image knowledge from discriminative feature representation, which is reflected by a discriminative feature representation based constraint terms and high quality prior image. This discriminative feature representation is realized using a composite dictionary containing both desirable tissue attenuation features and undesirable noise-artifacts residual features.

In this study, three sparse constraint based methods were evaluated, *i.e*., TV, GDSIR and the proposed PICCS_DFR_. The TV and PICCS_DFR_ methods can be regarded as voxel-wise updating methods while the GDSIR and DP-PICCS method are the patch-wise updating ones. Experiment results show that the PICCS_DFR_ method cannot achieve satisfactory performance in suppressing noise-induced artifacts as the indistinguishability true tissue structures and image noise-artifacts are significant, and, the GDSIR method cannot also effectively deal with the tiny tissue structures in LDCT reconstruction. The proposed DP-PICCS method obtains PSNR values 1-2 dB higher than the PICCS_DFR_ reconstruction, and the visual results also validate the improved ability of tissue structure discrimination. In the case that the projection data were seriously corrupted by excessive X-ray photon noise, the associative reconstructed images will suffer from serious noise-induced streak and mottle artifacts. In this case the DFR-post method cannot yield satisfactory results due to its ability in distinctive tissue and noise-artifacts structural.

The proposed DP-PICCS method can be feasibly implemented using the joint optimal reconstruction strategy in section II.B. However, due to the split alternating iteration scheme, the DP-PICCS method in sub-problem Eq. (6) and Eq. (7) are nonconvex for a global optimization. Similar to many existing dictionary learning based algorithms whose global convergence is an open issue, the proposed method also suffers from a lack of strict global convergence. Also, the DP-PICCS method needs to be accelerated to be more practically feasible. In the proposed approach, parameters were selected following the assumption that the same parameter setting can be used in the reconstructing the data from the same type of CT scanner. In^39^, a gradient projection method is applied in parameter selection where the regularization parameter is updated in an alternating mode. In^40^, a reweighted objective function is defined to guide the selection of the regularization parameters. These approaches can be applied to guide the parameter selection of the proposed DP-PICCS reconstruction^22,39,40^.

## References

[1] Schubert T (2012). CT-guided percutaneous biopsy of a mass lesion in the upper presacral space: A sacral transneuroforaminal approach. Cardiovasc. Intervent. Radiol..

[2] Siewerdsen JH (2011). Cone-beam CT with a flat-panel detector: From image science to image-guided surgery. Nucl. Instrum. Methods Phys. Res. A..

[3] Zhao B (2009). Evaluating variability in tumor measurements from same-day repeat CT scans of patients with non-small cell lung cancer. Radiology..

[4] Yu L (2009). Radiation dose reduction in computed tomography: Techniques and future perspective. Imag. Med..

[5] McCollough, C. H., Bruesewitz, M. R. & Kofler, J. M. Jr. CT dose reduction and dose management tools: Overview of available options. *Radiographics*. **26**, 503–512 (2006). 10.1148/rg.262055138 16549613

[6] Kalra MK (2004). Techniques and applications of automatic tube current modulation for CT. Radiology..

[7] Feldkamp L, Davis L, Kress J (1984). Practical cone-beam algorithm. JOSA A..

[8] Donoho DL (2006). Compressed sensing. IEEE Trans. Inf. Theory..

[9] Yu H, Wang G (2009). Compressed sensing based interior tomography. Phys. Med. Biol..

[10] Sidky E, Kao C, Pan X (2006). Accurate image reconstruction from few-views and limited-angle data in divergent-beam CT. J X-Ray Sci Tech..

[11] Sidky E, Pan X (2008). Image reconstruction in circular cone-beam computed tomography by constrained total-variation minimization. Phys. Med. Biol..

[12] Ritschl L (2011). Improved total variation-based CT image reconstruction applied to clinical data. Phys. Med. Biol..

[13] Tian Z (2011). Low-dose CT reconstruction via edge preserving total variation regularization. Phys. Med. Biol..

[14] Jia X (2011). Gpu-based iterative cone-beam CT reconstruction using tight frame regularization. Phys. Med. Biol..

[15] Garduño E, Herman G, Davidi R (2011). Reconstruction from a few projections by L1-minimization of the Haar transform. Inverse problems..

[16] Xu Q (2012). Low-dose X-ray CT reconstruction via dictionary learning. IEEE Trans. Med. Imag..

[17] Bai T (2014). 3D dictionary learning based iterative cone beam CT reconstruction. Int. J. Cancer Ther. Oncol..

[18] Chen Y (2014). Artifact suppressed dictionary learning for low dose CT image processing. IEEE Trans. Med. Imaging..

[19] Liu, J. *et al*. 3D Feature Constrained Reconstruction for Low Dose CT Imaging. *IEEE Trans. Circuits Syst. Video Technol*. (2016).

[20] Chen Y (2017). Discriminative feature representation: an effective post-processing solution to low dose CT imaging. Phys. Med. Biol..

[21] Caudevilla O, Brankov JG (2015). Learning based prior for analyzer-based phase contrast image reconstruction”. Biomedical Imaging (ISBI), 2015 IEEE 12th International Symposium on. IEEE..

[22] Mou X (2014). Dictionary learning based low-dose x-ray CT reconstruction using a balancing principle. Proc. SPIE 9212, Developments in X-Ray Tomography IX..

[23] Pfister, L. & Bresler, Y. Tomographic reconstruction with adaptive sparsifying transforms. *IEEE Int*. *Conf. Acoustics, Speech and Signal Processing(ICASSP)*, 6914–6918 (2014).

[24] Chen GH, Tang J, Leng S (2008). Prior image constrained compressed sensing (PICCS): a method to accurately reconstruct dynamic CT images from highly undersampled projection data sets. Med. Phys..

[25] Chen GH (2012). Time-resolved interventional cardiac C-arm cone-beam CT: An application of the PICCS algorithm. IEEE Trans. Med. Imag..

[26] Chen GH, Tang J, Hsieh J (2009). Temporal resolution improvement using PICCS in MDCT cardiac imaging. Med. Phys..

[27] Lauzier PT, Chen GH (2013). Characterization of statistical prior image constrained compressed sensing (PICCS)—Part II. Application to dose reduction. Med. Phys..

[28] Elbakri I, Fessler J (2002). Statistical image reconstruction for polyenergetic X-ray computed tomography. IEEE Trans. Med. Imaging..

[29] Elad, M. Sparse and Redundant Representations: From Theory to Applications in Signal and Image Processing. 121–350 (Springer, 2010).

[30] Elad M, Aharon M (2006). Image denoising via sparse and redundant representations over learned dictionaries. IEEE Trans. Image Process..

[31] Rubinstein R, Bruckstein AM, Elad M (2010). Dictionaries for sparse representation modeling. Proc. IEEE..

[32] Candes EJ, Eldar YC, Needell D, Randall P (2011). Compressed sensing with coherent and redundant dictionaries. Appl. Comput.Harmon. Anal..

[33] Goldstein T, Osher S (2009). The split Bregman method for L1-regularized problems. SIAM J. Imag. Sci..

[34] Chen SS, Donoho DL, Saunders MA (1998). Saunders. Atomic decomposition by basis pursuit. SIAM J. Sci. Comput..

[35] Rubinstein R, Zibulevsky M, Elad M (2008). Efficient implementation of the K-SVD algorithm using batch orthogonal matching pursuit. CS Technion..

[36] Wang Z, Bovik AC (2002). A universal image quality index. IEEE Signal Pro-cess Lett..

[37] Ravishankar S, Bresler Y (2011). MR image reconstruction from highly undersampled k-space data by dictionary learning. IEEE Trans. Med. Imaging..

[38] McCollough, C. The 2016 NIH-AAPM-Mayo Clinic Low Dose CT Grand Challenge. http://www.aapm.org/GrandChallenge/LowDoseCT/# (2016).

[39] Park JC (2012). Fast compressed sensing based CBCT reconstruction using Barzilai-Borwein formulation for application to on-line IGRT. Med. Phys..

[40] Zhang C (2015). A Model of Regularization Parameter Determination in Low-Dose X-Ray CT Reconstruction Based on Dictionary Learning. Computational and mathematical methods in medicine..

